# Integrative network-centric approach reveals signaling pathways associated with plant resistance and susceptibility to *Pseudomonas syringae*

**DOI:** 10.1371/journal.pbio.2005956

**Published:** 2018-12-12

**Authors:** Elizabeth K. Brauer, George V. Popescu, Dharmendra K. Singh, Mauricio Calviño, Kamala Gupta, Bhaskar Gupta, Suma Chakravarthy, Sorina C. Popescu

**Affiliations:** 1 The Boyce Thompson Institute for Plant Research, Ithaca, New York, United States of America; 2 Institute for Genomics, Biocomputing, and Biotechnology, Mississippi State University, Mississippi State, Mississippi, United States of America; 3 The National Institute for Laser, Plasma & Radiation Physics, Bucharest, Romania; 4 Department of Plant Pathology, Cornell University, Ithaca, New York, United States of America; 5 Department of Biochemistry, Molecular Biology, Entomology and Plant Pathology, Mississippi State University, Mississippi State, Mississippi, United States of America; The Sainsbury Laboratory, United Kingdom of Great Britain and Northern Ireland

## Abstract

Plant protein kinases form redundant signaling pathways to perceive microbial pathogens and activate immunity. Bacterial pathogens repress cellular immune responses by secreting effectors, some of which bind and inhibit multiple host kinases. To understand how broadly bacterial effectors may bind protein kinases and the function of these kinase interactors, we first tested kinase–effector (K-E) interactions using the *Pseudomonas syringae* pv. *tomato*–tomato pathosystem. We tested interactions between five individual effectors (HopAI1, AvrPto, HopA1, HopM1, and HopAF1) and 279 tomato kinases in tomato cells. Over half of the tested kinases interacted with at least one effector, and 48% of these kinases interacted with more than three effectors, suggesting a role in the defense. Next, we characterized the role of select multi-effector–interacting kinases and revealed their roles in basal resistance, effector-triggered immunity (ETI), or programmed cell death (PCD). The immune function of several of these kinases was only detectable in the presence of effectors, suggesting that these kinases are critical when particular cell functions are perturbed or that their role is typically masked. To visualize the kinase networks underlying the cellular responses, we derived signal-specific networks. A comparison of the networks revealed a limited overlap between ETI and basal immunity networks. In addition, the basal immune network complexity increased when exposed to some of the effectors. The networks were used to successfully predict the role of a new set of kinases in basal immunity. Our work indicates the complexity of the larger kinase-based defense network and demonstrates how virulence- and avirulence-associated bacterial effectors alter sectors of the defense network.

## Author summary

Some bacterial pathogens secrete virulence factors called effectors, which influence host tissues during infection. The impact of such bacterial effectors on the transmission of immune signals in plants remains poorly understood. In this study, we developed an integrative network approach to discover interactions between bacterial effectors and a class of host signal-mediating enzymes called protein kinases. We also characterized the functions of the targets of these kinases in order to understand how bacterial effectors might disrupt the flow of information in signaling pathways within plant cells. We show that plants activate larger signaling networks when inoculated with pathogens that produce effectors. We also find that plant signaling networks are specific to individual effectors and that the networks include kinases with both positive and negative effects on plant resistance to pathogens. We propose that the topology of immune signaling networks is determined by the plant’s ability to activate compensatory pathways in response to the effectors’ network-disruptive actions. Conversely, pathogens may increase their virulence both by disrupting host signaling at the membrane-located end of the signaling network and by recruiting cytosolic kinases. This work provides a framework for the study of plant–pathogen communication and could be used to prioritize targets for improving resistance in crops.

## Introduction

Plant immunity is generated by the activation and coordination of several protein kinase-based signal transduction pathways into cellular defense responses [1,2]. Kinases modify the activity status of other proteins through specific biochemical modifications (substrate phosphorylation) or by recruiting proteins in signaling complexes. Signaling pathways transmit pathogen signals from the cell periphery to intracellular compartments and trigger changes in gene expression, hormone-based signaling, and defense compound production [3]. To survive in plant tissues and ensure spread to other plants, pathogens must overcome plant defenses and redirect their energetic and nutrient resources.

The constant tug-of-war between plants and pathogens has generated a complex immune system in plants, and equally multifaceted assault and endurance mechanisms in pathogens. Plant pathogens such as the gram-negative flagellated bacterium *Pseudomonas syringae* can colonize a broad range of plants, an ability at least partly determined by an extensive and versatile effector repertoire [4, 5]. *P*. *syringae* subverts the basal immunity in part by attacking components of signaling pathways activated by pathogen-associated molecular patterns (PAMPs) or secreted effectors. PAMP-triggered immunity (PTI) is induced by PAMP perception by pattern recognition receptors (PRRs), some of which are receptor-like kinases (RLKs). Upon PAMP recognition, PRRs activate membrane-associated receptor-like cytosolic kinases (RLCKs), cytosolic mitogen-activated protein (MAP) kinase (MAPK) cascades, and other cytosolic kinases, including Ca^2+^-dependent kinases [6]. Effector-triggered immunity (ETI), the second layer of immunity, is activated by direct or indirect recognition of effectors, followed by activation of signaling pathways and induction of defense responses and programmed cell death (PCD). However, most intracellular effectors are not recognized by the plant and instead are thought to impair the plant’s ability to sustain an efficient immune response, a condition described as effector-triggered susceptibility (ETS) [7].

Work with *Arabidopsis*, tomato (*Solanum lycopersicum*), and *Nicotiana benthamiana* has identified specific *P*. *syringae* effectors that inactivate plant kinases [8, 9]. For example, the AvrPto effector binds membrane-associated kinases, including the PRRs FALGELLIN-SENSING2 (FLS2), EF-TU RECEPTOR (EFR), and the BRI1-ASSOCIATED RECEPTOR KINASE1 (BAK1) co-receptor to disrupt PTI and promote bacterial virulence [10]. The AvrPto effector also induces host resistance in some tomato genotypes by interacting with the Pto kinase, which activates Pseudomonas resistance and fenthion sensitivity (Prf) resistance protein, resulting in ETI [11]. Another effector called HopAI1 represses PTI through its interactions with the cytosolic MAPKs, MPK3 and MPK6 [12]. Recent work suggests that pathogen effectors may interact with not only a few targets but with multiple host targets, indicating that the breadth of effector–plant interactions are only beginning to be understood. Proteome-scale interactomics [13, 14] revealed an impressive number of putative effector-interacting proteins alongside fundamental properties of plant–pathogen interaction networks, such as effector convergence on network hubs. Furthermore, using a different methodology (i.e., global transcriptional profiling of *Arabidopsis* defense-related mutants coupled with modeling) [15] identified regulatory relationships between immune-related subnetworks and highly interconnected network components. However, in these studies, the physical layout of the underlying plant cellular networks targeted by pathogens remained out of the reach of the analytic and experimental methodologies utilized.

To better understand how protein kinases contribute to basal immunity, we first sought kinase targets that interact with multiple effectors, a characteristic of defense-associated host proteins [13, 14]. Five *P*. *syringae* effectors (AvrPto, HopA1, HopAI1, HopAF1, and HopM1) were selected based on two main criteria: high prevalence among the *P*. *syringae* isolates [5] and a known ability or potential to suppress defense responses [10, 16–19]. HopA1 disrupts the formation of a protein complex involved in activating basal immunity and ETI [20]. HopAF1 interacts with the methylthioadenosine nucleosidase proteins MTN1 and MTN2 to disrupt ethylene (ET) production [18]. HopM1 binds HopM1 interactor 7 (MIN7), disrupting vesicle trafficking and reducing callose deposition [19]. These five effectors suppress different parts of the cellular immune response in plants, suggesting that they may interact with distinct host proteins. Here, we identify targets of bacterial effectors in plant cells, perform an in-depth functional analysis of a set of multi-effector–interacting kinases to superimpose effector-specific pathways over the plant–effector interaction space, and characterize the properties of the plant defense network.

## Results

### An integrated approach to plant–pathogen molecular communication

**Fig 1:**
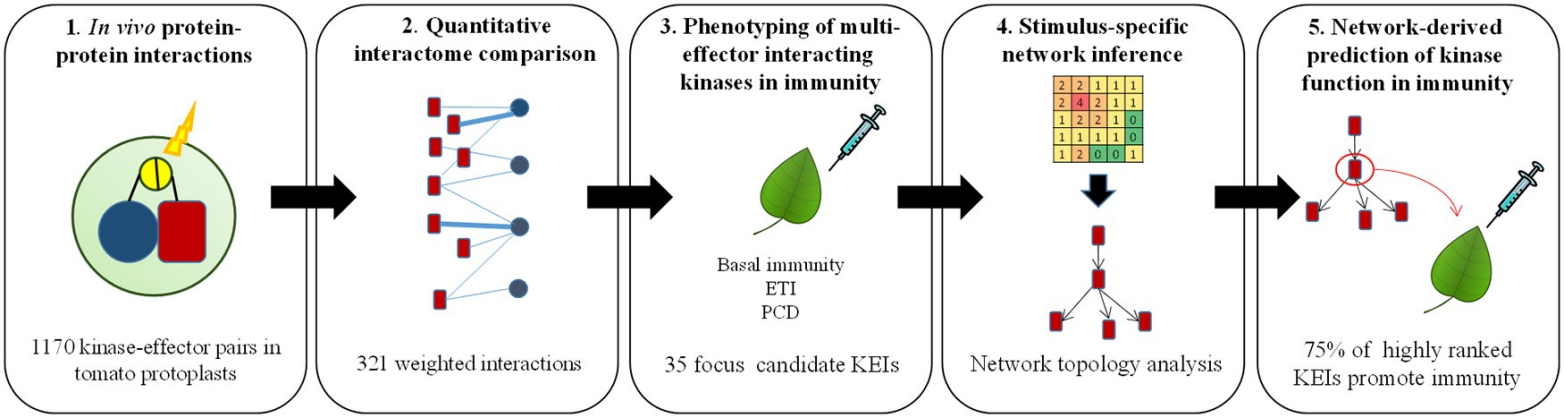
The pipeline of the integrative approach to study plant immune system. Pairwise interactions between a library of 279 tomato kinases and five *Pseudomonas syringae* effectors were tested using a reporter-based luminescence assay in tomato protoplasts, with readings at six time points. This data set was the basis of a quantitative K-E interaction network generated for 321 significant interactions comprising 133 kinases (KEIs) and four of the tested effectors (1). Thirty-five multi-effector *KEIs* were selected for further analyses in planta (2). The functions and quantitative contributions of the focus *KEIs* were tested in several plant phenotypes: PTI, ETS, ETI, and MAPK-mediated PCD (3). These data sets were combined in a *KEI* phenotype co-occurrence matrix that was expanded into a composite immune/cell death, directed signaling network, in which modules and pathways were rank ordered, and stimulus-specific networks were inferred (4). Highly ranked nodes were tested for involvement in immune responses and over 75% of the highly ranked kinases were involved in immunity (5). ETI, effector-triggered immunity; ETS, effector-triggered susceptibility; K-E, kinase–effector; KEI, Kinase Effector Interactor; MAPK, MAP kinase; PCD, programmed cell death; PTI, PAMP-triggered immunity.

We developed a multipronged approach consisting of identification of in vivo pairwise interactions between 279 tomato kinases and five effectors from the model tomato pathogen, *Pseudomonas syringae* pv. *tomato (Pst)* (HopA1, HopAI1, HopAF1, AvrPto, and HopM1). Next, we characterized the role of 35 multi-effector–interacting kinases in PTI, ETS, ETI, and PCD. We created new methodologies for data integration and generated signaling networks to facilitate visualization of the protein kinase networks involved in defense (Fig 1). This network-centric approach allowed us to compare signaling networks associated with different levels of plant immunity and led to identification of novel defense-associated kinases. The approach and the results obtained are described in the sections that follow and in the Supporting information (S1 Materials and Methods).

### An in vivo interaction screen of tomato kinases and *P*. *syringae* effectors reveals known and novel putative interactions

To better understand how diverse effectors may be targeting plant protein kinases, we tested interactions between 279 tomato kinases [21] and five effectors. A total of 1,170 pairwise kinase–effector (K-E) interactions were tested in tomato protoplasts using the split luciferase complementation assay (SLCA), where luciferase activity indicates reconstitution of the N-terminal and C-terminal domains of the enzyme fused to interacting bait or prey proteins. The split luciferase complementation (SLC) data analysis is described in the Supporting information (S1 Materials and Methods). Interactomics primary data are available at https://figshare.com/s/35c4aab65174c67a496e); the MATLAB code for the SLC data analysis is provided in S3 Data. Several controls were included in each SLC experiment to ensure reproducibility of the method across replicates, including a positive control (protoplasts with full-length luciferase), a negative control (untransformed protoplasts), and a reference interaction set between the AvrPto effector and the Pto kinase, which have been shown to interact in planta [22]. In addition, an AvrPto^I96A^ (AvrPto with an Ile to Ala mutation) and Pto kinase pair were included as a control for interaction strength because the I96A mutation inhibits effector function and interaction with the Pto kinase [10]. The SLCA screen was reproducible with low variability of luminescence signals across technical replicates (S1A Fig). High correlation was observed for the signals for full-length luciferase and controls (reference set of positive and negative interactions) among plates (S1B Fig); the K-E signals and the control sets did not show correlation, indicating a lack of a measuring bias in the protocol (S1C Fig). The signals from the AvrPto–Pto and AvrPto^I96A^–Pto interactions were highly correlated with an average 3-fold reduction in signal for the AvrPto^I96A^–Pto interaction. A multiple regression model of Pto–AvrPto^I96A^ versus Pto–AvrPto and Luciferase signals has *R*^2^ = 0.887 (adjusted *R*^2^ = 0.886) and regression coefficients of 7.17 × 10^−3^ (Luciferase) and 3.06 × 10^−1^ (Pto–AvrPto) (S1D Fig). Moreover, the luminescence signal of the K-E pairs showed a wide dynamic range, indicating that there are no physical limitations in measuring the luminescence produced in the SLCA (S1E Fig).

**Fig 2:**
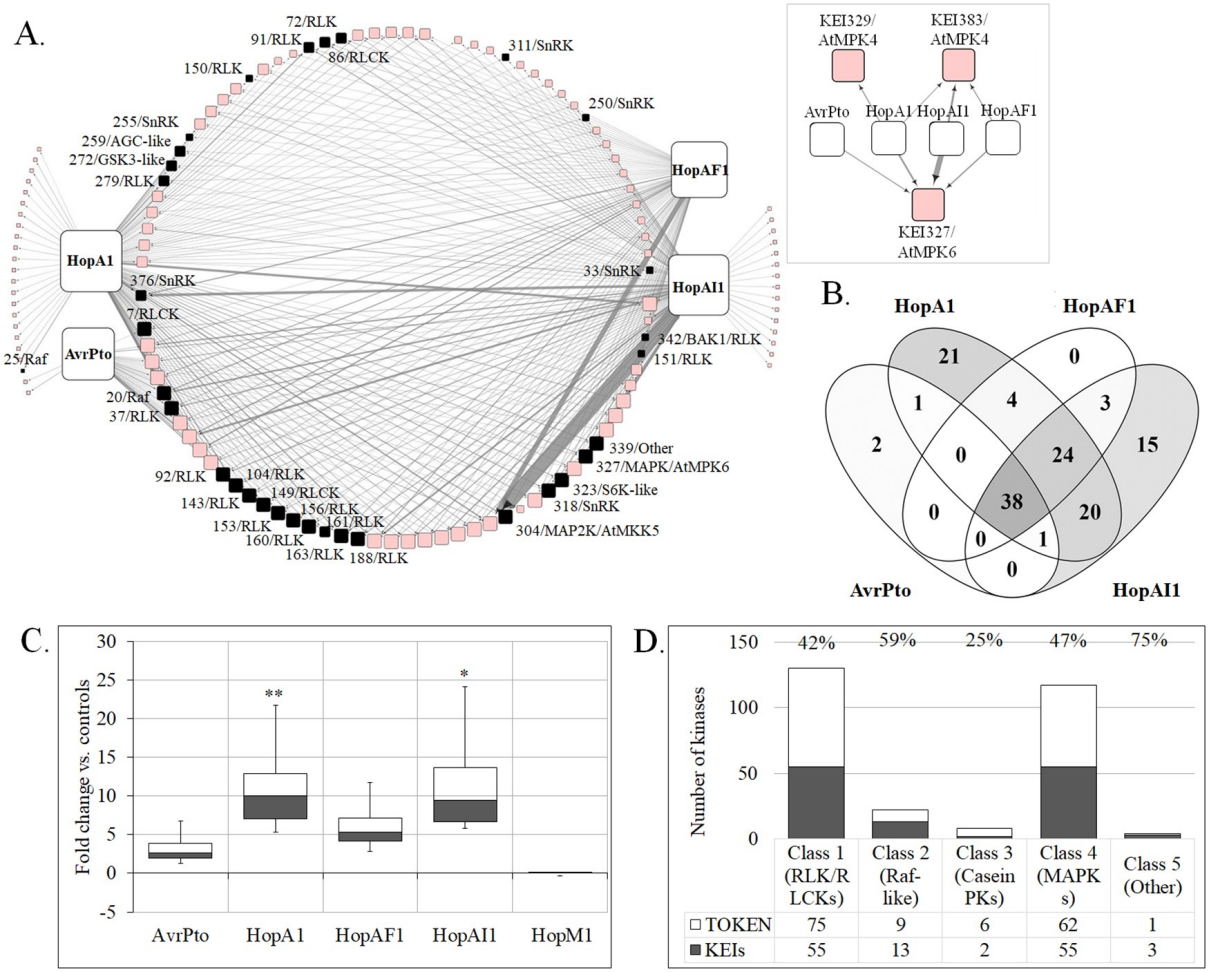
A plant–pathogen interaction screen identifies new candidate plant targets. **A.** An in vivo interaction network of tomato kinases with *Pseudomonas syringae* effectors. We tested 210 Kinase–AvrPto pairs, 299 Kinase–HopA1, 305 Kinase–HopAI1, and 326 Kinase–HopAF1 using SLC in tomato cells. The node size represents degree (number of outgoing and incoming edges; larger size = higher degee), and edge thickness indicates the interaction strength (fold increase over control) on a continuous scale (thicker = stronger interaction). Known K-E interactions tested in the SLCAs are represented separately in the inset network. The inset network shows interactions of the tomato homologs of the known effector targets *KEI327/SlMPK1* and *KEI384/AtMPK4*, with four effectors. Nodes in black represent the KEIs selected for functional characterization. The networks were visualized in Cytoscape 3.6.1. **B.** Venn diagram showing common and unique interacting kinases for HopA1, HopAF1, HopAI1, and AvrPto. **C.** Boxplots showing the distribution of interaction strength represented as fold change versus controls, for the five individual effectors tested. *p < 0.01 or **p < 0.05, *t* test. **D.** The distribution of KEIs across the five structural classes of plant kinases. The digits show the percentage of KEIs found to interact with pathogen effectors within each kinase class. BAK1, BRI1-ASSOCIATED KINASE1; K-E, kinase–effector; KEI, Kinase Effector Interactor; MAPK, MAP kinase; MAP2K, MAPK kinase kinase; PK, protein kinase; RLCK, receptor-like cytosolic kinase; RLK, receptor-like kinase; SLC, split-luciferase complementation; SLCA, split-luciferase complementation assay; SnRK, Snf1-related protein kinase; S6K, ribosomal protein S6 kinase; TOKN, Tomato Kinase cDNA library.

Out of the 279 kinases tested for interactions with AvrPto, HopA1, HopAI1, or HopAF1, 133 (48%) interacted with at least one effector and were named Kinase Effector Interactors (KEIs). No significant interactions were identified for HopM1 out of the 30 tested kinases, suggesting that this endomembrane-specific effector [19] may not associate with kinases. The K-E interaction network contains 321 significant interactions of 133 kinases with four effectors and includes previously confirmed K-E interactions (Fig 2A; S1 Table). Among the 133 kinases, approximately 70% are multi-effector interactors, out of which 38 interact with all four effectors and 24 are shared by HopA1, HopAI1, and HopAF1 (Fig 2B). To estimate the relative affinity of K-E interactions, a metric called the “interaction strength coefficient” (normalized signal fold change) was used to quantify the difference in reconstituted luciferase activity between each tested pair and the reference interactions. On average, the interactions of KEIs with HopA1 or HopAI1 were twice as strong when compared with AvrPto, possibly due to the better reconstitution of the luciferase, higher affinity, or low dissociation of K-E complexes (Fig 2C). Notably, two known interaction pairs (HopAI1–MPK6 and HopAI1–MPK4) were the strongest among all control interactions tested for these MAPKs (inset of Fig 2A). The distribution of the fold change interaction values off all K-E interactions tested is shown in S2A Fig. An analysis of the candidate *KEIs* along the spectrum of kinase classes [23] revealed that the effectors interacted mostly with leucine-rich repeats (LRR)-type RLKs and RLCKs from Class 1 (42%), kinases from Class 2/Raf-like (59%), and Class 4/MAPKs and calcium-responsive kinases (47%) (Fig 2D; S2B Fig).

### RLKs and RLCKs promote basal immunity, while cytosolic kinases promote ETS

**Fig 3:**
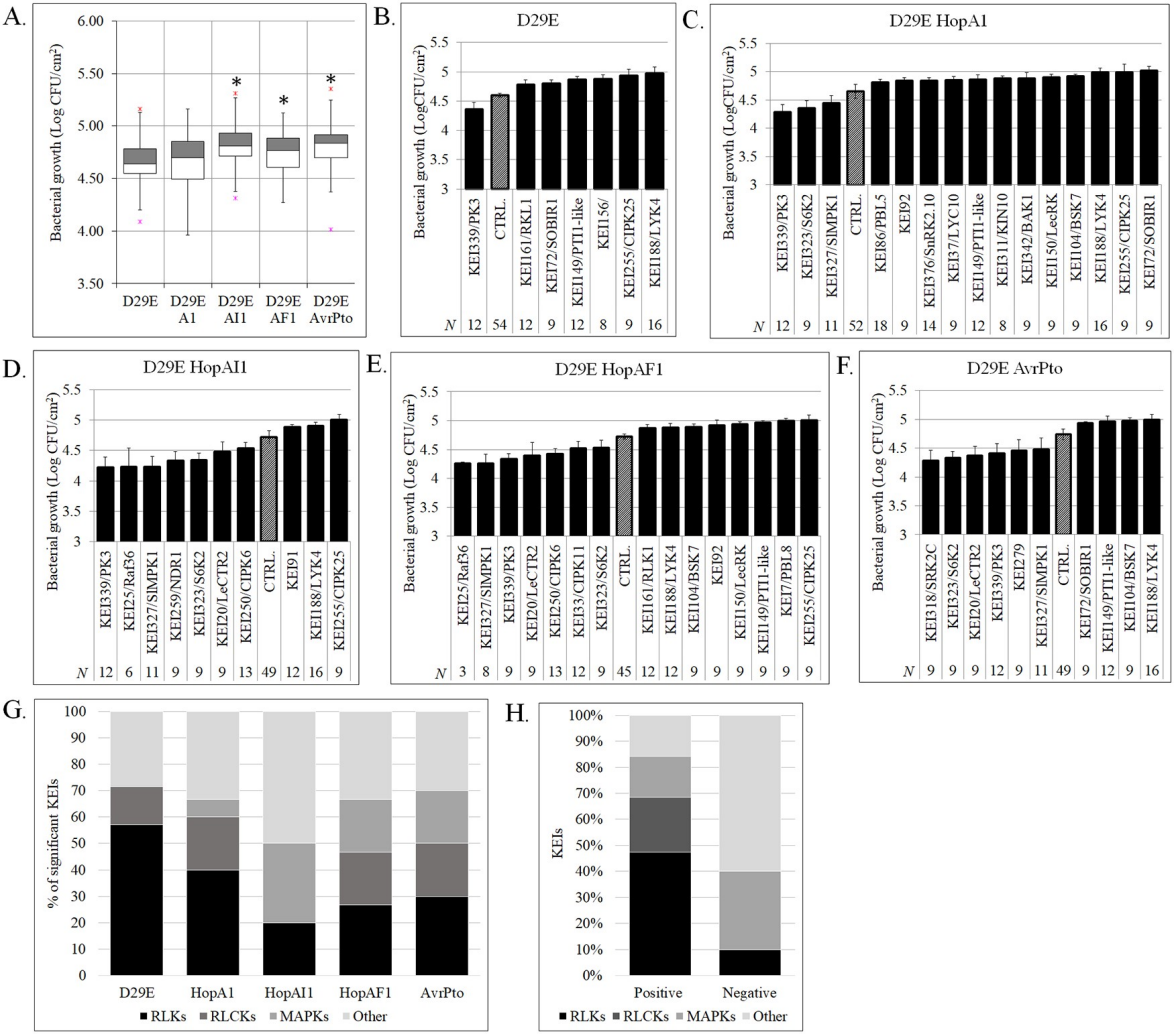
Plant kinases promote defense or susceptibility to *Pseudomonas syringae*. **A.** Quantification of *P*. *syringae* growth without effectors (D29E) or with one effector (D29E + HopA1, D29E + HopAI1, D29E + HopAF1, or D29E + AvrPto) in *Nicotiana benthamiana* leaves transformed with the EC1 control plasmid. Statistically significant events (*) are considered at *p* < 0.01 compared with D29E. **B–F.** Quantification of the growth of *P*. *syringae* strains in *N*. *benthamiana* leaves silenced for individual kinases (*KEIs*), where only *KEIs* with statistically significant (*p* < 0.05) phenotypes were plotted. The control values (CTRL.) represent quantification of the *P*. *syringae* growth in plants silenced by the EC1 control. **G.** Histogram with the percentage and structural class of *KEIs* identified as regulators of pathogen growth in plant tissues inoculated with the *P*. *syringae* strains D29E, D29E + HopA1, D29E + HopAI1, D29E + HopAF1, and D29E + AvrPto. **H.** Histogram with the number of KEIs shown to act as candidate positive or negative regulators of immunity based on all infection assays. CFU, colony-forming unit; *KEI*, *Kinase Effector Interactor*; MAPK, MAP kinase; *N*, number of independent replicates; RLCK, receptor-like cytosolic kinase; RLK, receptor-like kinase.

A group of *KEIs* was selected for functional characterization based on their ability to putatively interact with multiple effectors. The 35 focus *KEI*s (S2 Table) were silenced in *N*. *benthamiana*, a relative to tomato and host for *Pseudomonas syringae* DC3000 strains lacking the HopQ1-1 avirulence gene, due to its amenability for transformation and high efficiency of gene silencing [24, 25]. Virus-induced gene silencing (VIGS) constructs containing a fragment of an *Escherichia coli* gene (EC1) served as a negative control. After confirmation that *KEI* expression was silenced, the plants were inoculated with an effectorless *Pst* strain (D29E) [26] and four single-effector strains expressing *AvrPto*, *HopA1*, *HopAF1*, or *HopAI1* in the D29E background (S3 Table; S3A Fig; S1 Data). In the *EC1* control, the presence of some effectors (*AvrPto*, *HopAI1*, or *HopAF1*, but not *HopA1*) in D29E led to a moderate but significant increase in *Pst* growth compared to D29E (Fig 3A), indicating that these effectors can contribute to *Pst* virulence in isolation from the broader repertoire. Among the 35 *KEI*s, seven *KEIs* influenced D29E growth compared with the *EC1* control, indicating a role in basal immunity (Fig 3B**)**. The majority of these *KEIs*—including *RLKs* (*KEI188/LYK4*, *KEI72/SOBIR1*, *KEI156*, and *KEI161/RKL1)*, *RLCKs (KEI149/PTI1*-like), and the Ca^2+^-regulated *KEI255/CIPK25*—promoted bacterial growth when silenced, while silencing of one kinase (*KEI339*) inhibited D29E growth. In comparison, silencing of 17 *KEIs* caused a significant change in the growth of single-effector strains compared with the EC1 control (Fig 3C–3F; S3B and S3C Fig). Silencing of *SOBIR1*, a key component of PTI [27, 28], affected the growth of D29E and the HopA1- and AvrPto-carrying strains. Moreover, silencing of *KEI342*/*SlBAK1* (one of the tomato *BAK1* homologs that may facilitate basal immunity [29]) or of *KEI327*/*SlMPK1* (a kinase with high similarity to *AtMPK6* and a possible role in PTI [30]) interfered with the growth of single-effector strains exclusively.

Interestingly, the majority of *KEIs* required for *D29E* response were *RLKs*. On the other hand, cytosolic kinases were preponderant in plant response to D29E + HopAI1, + HopAF1 or + AvrPto, showing a 4-, 2.8-, and 2.3-fold increase, respectively, relative to the *RLKs* (Fig 3G). Among the *KEIs* with significant contributions to bacterial growth, 52% participated in plant response to D29E + HopA1 and + HopAF1 and 36% to D29E + AvrPto and HopAI1, while only 12% were necessary for defense against D29E (S3D Fig). *KEIs* had significant positive or negative effects on the growth of *Pst* strains, indicating that *KEIs* promote either immunity or ETS, but not both (S3E Fig). Overall, when mapping the sign of variation (Fig 3H), most *KEIs* classified as *RLK/RLCKs* promoted basal immunity, while *KEIs* promoting ETS mainly included kinases from the other cytosolic and *MAPK*-like (60% and 30%, respectively).

### Diverse classes of *KEIs* facilitate ETI, ETS, and MAPK-mediated PCD

**Fig 4:**
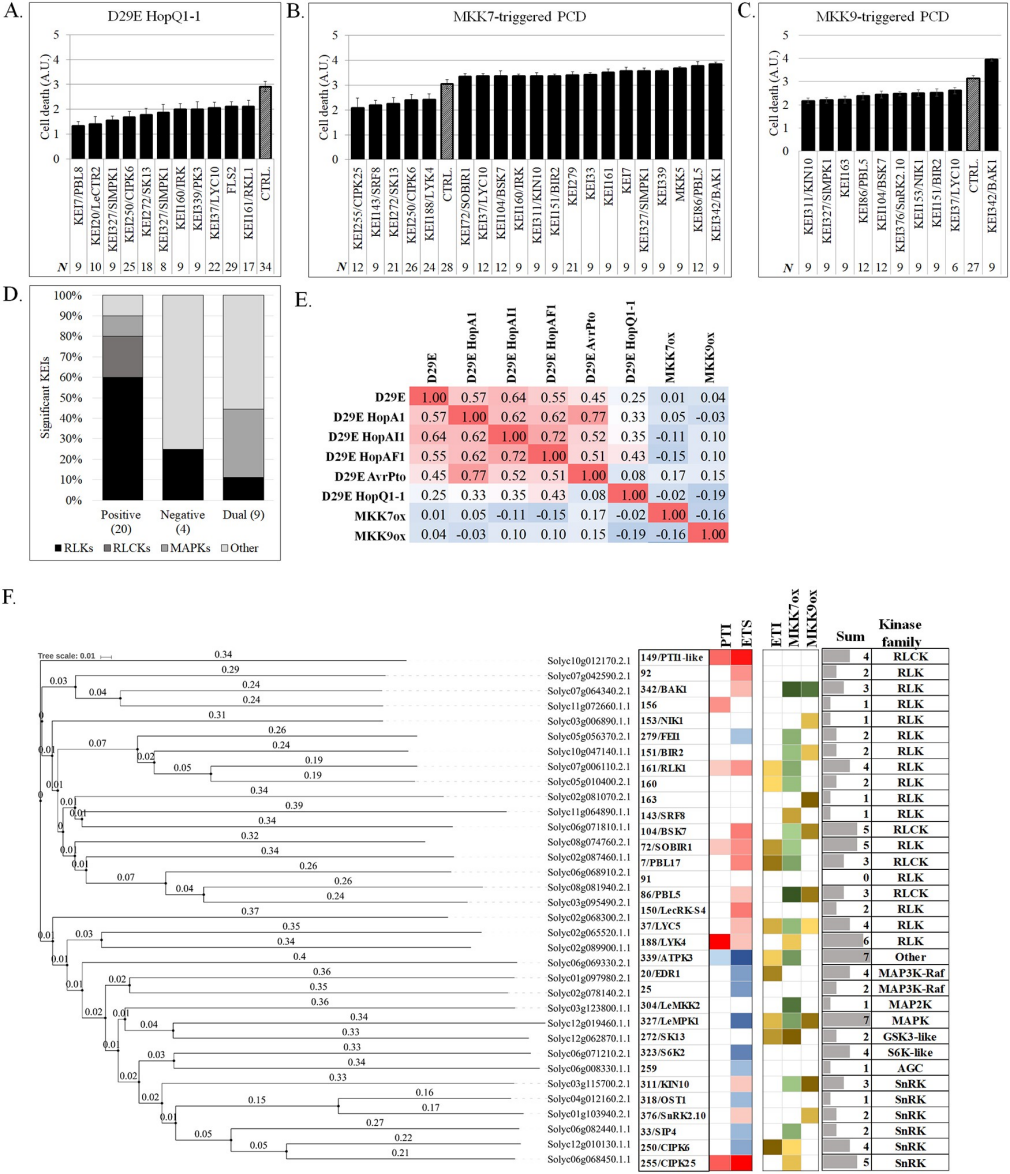
Kinases promote or inhibit cell death associated with the ETI or prolonged activation of MAPKs. **A–C.** Quantification of the PCD intensity in *Nicotiana benthamiana KEI*-silenced or EC1 control plants (CTRL) infiltrated with the ETI-inducing strain *Pseudomonas syringae* D29E + HopQ1-1 (A), or following overexpression of constitutively active MKK7 or MKK9 (B and C). Cell-death intensity was measured as described in Materials and methods and presented on a scale from 0 to 4 artificial units (A.U.’s). **D.** Histogram showing the percentages of *KEI*s with a positive, negative, or dual regulatory effect on cell death across kinase classes, including *RLKs*, *RLCKs*, *MAPKs*, and other cytosolic kinases. The number of kinases in each regulatory category is shown in parentheses. **E.** A heat map of the Pearson correlation coefficients (*R*) calculated for pairwise combinations of the eight orthogonal assays testing the defense and cell death responses in silenced plants. The map reveals similarities in *KEIs’* importance for the plant response to effectorless, single-effector *P*. *syringae* strains, and cell death treatments. **F**. Phylogram of tomato *KEIs* shown alongside a phenotype heat map matrix of *KEIs* strength of regulation (−log_10_ of *p*-value) in bacterial inoculation assays (red: *KEIs* positive regulators defense; blue: *KEIs* positive regulators of susceptibility) and cell death–inducing treatments (brown: *KEIs* promoting cell death; green: *KEIs* inhibiting cell death). The sum column indicates the number of assays (minimum = 1 and maximum = 8) for which *KEIs* exhibited statistically significant phenotypes. The kinase family information is shown on the right. A.U., artificial unit; BAK1, BRI1-ASSOCIATED KINASE1; ETI, effector-triggered immunity; ETS, effector-triggered susceptibility; *KEI*, *Kinase Effector Interactor*; MAPK, MAP kinase; MKK, MAP kinase kinase; PCD, programmed cell death; PTI, PAMP-triggered immunity; RLCK, receptor-like cytosolic kinase; RLK, receptor-like kinase.

Some protein kinases have been shown mediate cellular response to multiple types of stresses [31]. To determine if *KEIs* are similarly involved in multiple response pathways, we tested the focus *KEIs* in ETI and MAPK-mediated PCD responses. The *HopQ1-1* effector is an avirulence factor in *N*. *benthamiana*, in which it is recognized by an unknown R protein [32]. To test the ETI in *KEI*-silenced plants, we quantified the size of the necrotic lesion [32] triggered by the inoculation with a D29E + *HopQ1-1* strain as a proxy for quantification of PCD (S4A Fig; S1 Data**)**. Eleven out of the 35 tomato *KEIs* tested were required for PCD, including *RLKs* (*KEI37/LYC10*, *KEI161/RKL1*, and *AtFLS2*), *RLCKs* (*KEI7*/*PBL8*), *MAPKK kinases MAP3Ks* (*KEI20/SlCTR1*), *SnRKs* (*KEI250/CIPK6*), *GSK3/Shaggy*-like (*KEI272/SK13*), and *KEI339* (Fig 4A; S4B Fig). Silencing of *KEI72*/*SOBIR1*, a known positive cell death modulator [33], *KEI7/PBL8*, and *KEI20/SlCTR1* impaired PCD the most. *KEI160/NtIRK*, previously associated with antiviral defense and regulation of the R-gene–mediated PCD in *N*. *benthamiana* [34], facilitated PCD. The results indicate that many *KEIs* mediate ETI-associated PCD by exerting exclusively positive regulatory roles.

To test the role of *KEIs* in the development of MAPK-dependent PCD, *KEI*-silenced plants were infiltrated with constitutively active MKK7 or MKK9. Both MAPK kinases (MAP2Ks) are known to participate in multiple immune-related processes [35, 36], and their prolonged expression induces activation of MPK3 and MPK6 and PCD [1]. Lesion size was significantly altered in 23 of the tested *KEI*-silenced lines following *MAP2K* expression. *MKK7*-triggered, *MKK9*-triggered PCD was modified in several lines, seven of which were required for both *MKK7*- and *MKK9*-mediated pathways (Fig 4B and 4C). The *RLKs* and *RLCKs* functioned as both positive and negative PCD regulators, compared with other classes (Fig 4D). Notably, silencing of the PCD negative regulator *KEI342/BAK1* [37] inhibited both *MKK7*- and *MKK9*-PCD.

To determine how these phenotypes may be related, we performed correlation analyses between bacterial growth and lesion size measurements across the *KEI* lines (Fig 4E), as described in S1 Materials and Methods. A significant correlation (*R* > 0.6) was observed across bacterial growth assays, but no correlation was found between bacterial growth and PCD treatments, suggesting these responses utilize distinct signaling pathways. To obtain a global view of the link between the *KEIs*’ structural class and their contribution to immune phenotypes, we plotted a phylogenetic tree and visualized significant contributions to immunity based on our assays (Fig 4F and S2 Data). Interestingly, the phylogenetic tree highlighted the clear difference in structural class between the PTI- versus ETS-promoting kinases (*RLKs*/*RLCKs* versus MAPKs, *CIPK/SnRKs*, *ribosomal protein S6 kinase* (*S6K*), *AGC kinases*, and *glycogen synthase kinase3* [*GSK3*]-like, respectively).

### Effectors manipulate defense network topology, increasing complexity of the network

**Fig 5:**
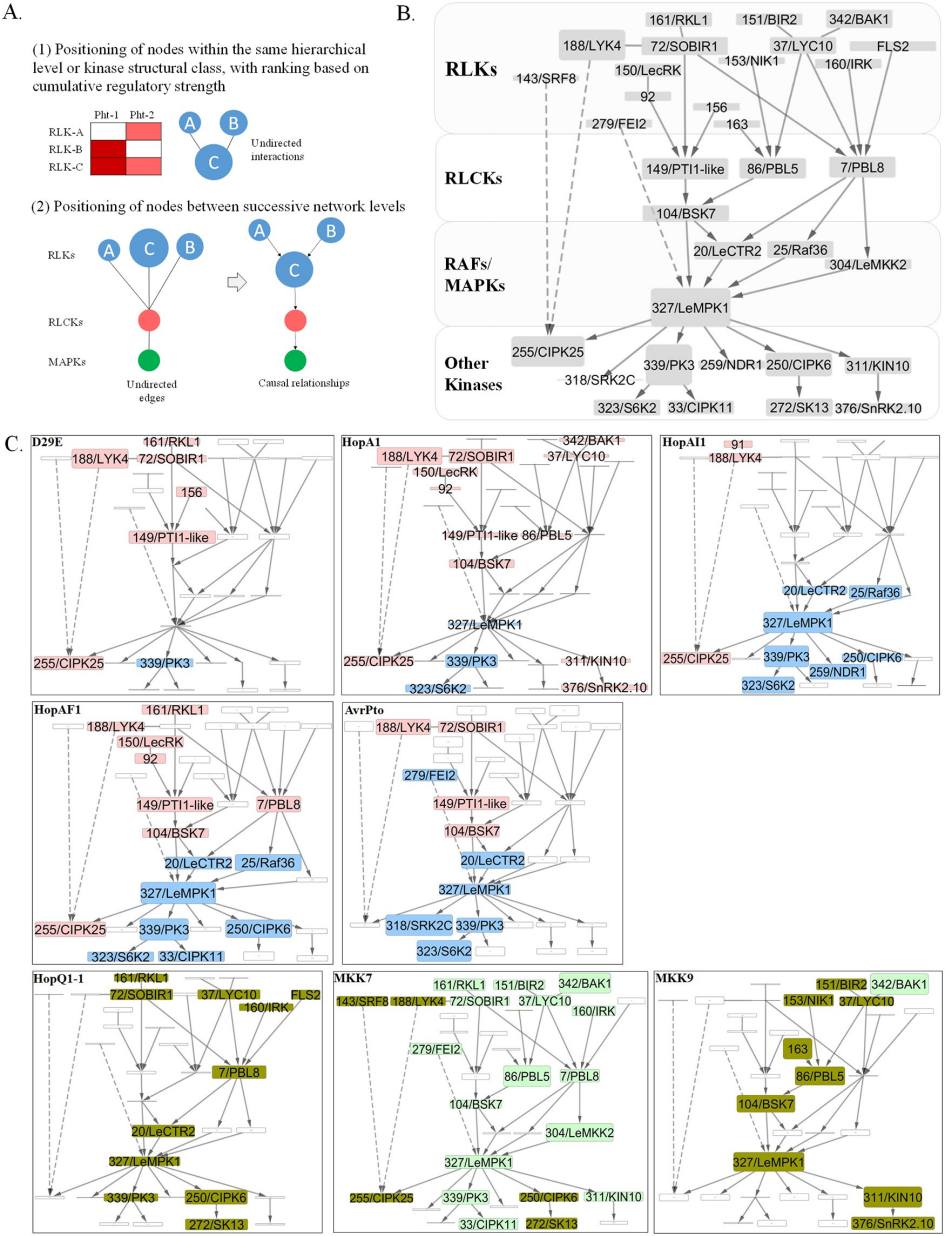
Inference of stimulus-specific *KEI* genetic networks. **A.** Schematic diagram for deriving causal relationships in the *KEI* genetic network. Associations between *KEIs* were calculated based on phenotyping assays using the typical hierarchical topology of a signal transduction network (RLK → RLCK → MAPK/RAF → other kinases). The main criteria for assembling the genetic pathways were (1) assembly of modules based on co-occurrence of the *KEI* within the same level or kinase structural class and (2) linkage of *KEI*s between network levels, based on co-occurrence, while minimizing redundant edges. Two additional secondary criteria were used: *KEI p*-values (see S3C and S4B Figs) determined position within the modules/pathways (where high-significance *KEIs* are placed in hub positions as high-degree nodes), and the *KEI*’s sign of regulation (same-sign *KEIs* placed in same module/pathway). **B.** Hierarchical weighted network representing the collapsed (minimal) network of *KEIs* with statistically significant phenotypes in immunity and ETI or PCD. Nodes represent *KEIs* and node size is proportional to the cumulative significance values (see S3C and S4A Figs); higher diameter indicates a lower *p*-value. The four hierarchical levels of the network, including the *KEIs* within each level, are shown. **C.** Visualization of stimulus-specific networks using the same structure as shown in (B). Individual networks indicate responses to distinct *Pseudomonas syringae* strains and cell death–inducing treatments to enable comparison of networks across stimuli. Node height is proportional to regulatory strength (*p*-value), and node color indicates significance of the node in promoting immunity (red), promoting susceptibility (blue), and repressing cell death (green) or promoting cell death (olive). Edges are nontargeted, representing interaction events (known or predicted), or targeted to show the signal flow within the network. Edges with broken lines show an atypical directionality that bypasses one or several levels within the signaling network. The position of several *KEIs* within the network could not be unambiguously determined (*KEI143*, *KEI188*, *KEI255*, and *KEI279*), and their associations are shown with broken edges that skip over hierarchical network levels. ETI, effector-triggered immunity; *KEI*, *Kinase Effector Interactor*; MAPK, MAP kinase; MKK, MAPK kinase; PCD, programmed cell death; RLCK, receptor-like cytosolic kinase; RLK, receptor-like kinase.

The involvement of *KEI*s in multiple stress responses prompted the development of functional signaling networks to understand how defense networks are modified during different immune responses. To construct the networks, we calculated the co-occurrence frequency of the focus *KEIs* in various functional assays to evaluate the degree of *KEIs* phenotype overlap, indicative of functional association among *KEIs*. Using a set of logical rules and prior information (Fig 5A), a KEI signaling network was generated with the nodes (*KEIs*) ordered hierarchically within the canonical structure of a signaling pathway: RLK → RLCK → RAFs/MAPKs → cytosolic kinases (Materials and methods; S1 Materials and Methods). Indirect evidence positioned RAFs upstream of MAPK cascades and at a similar hierarchical level with MAP2Ks [2, 31, 38, 39]. Directed edges weighted by co-occurrence values link the nodes. To generate the signaling network, *KEIs* with similar phenotypes were grouped in modules, in which the position of nodes within the same hierarchical level or kinase structural class was based on their regulatory strength rank and sign of regulation, while the redundant edges between successive network levels were removed. Using these criteria, we collapsed the weighted composite graphs into a minimal network providing an overview of the *KEI* pathways critical to immune-related plant phenotypes (Fig 5B).

**Fig 6:**
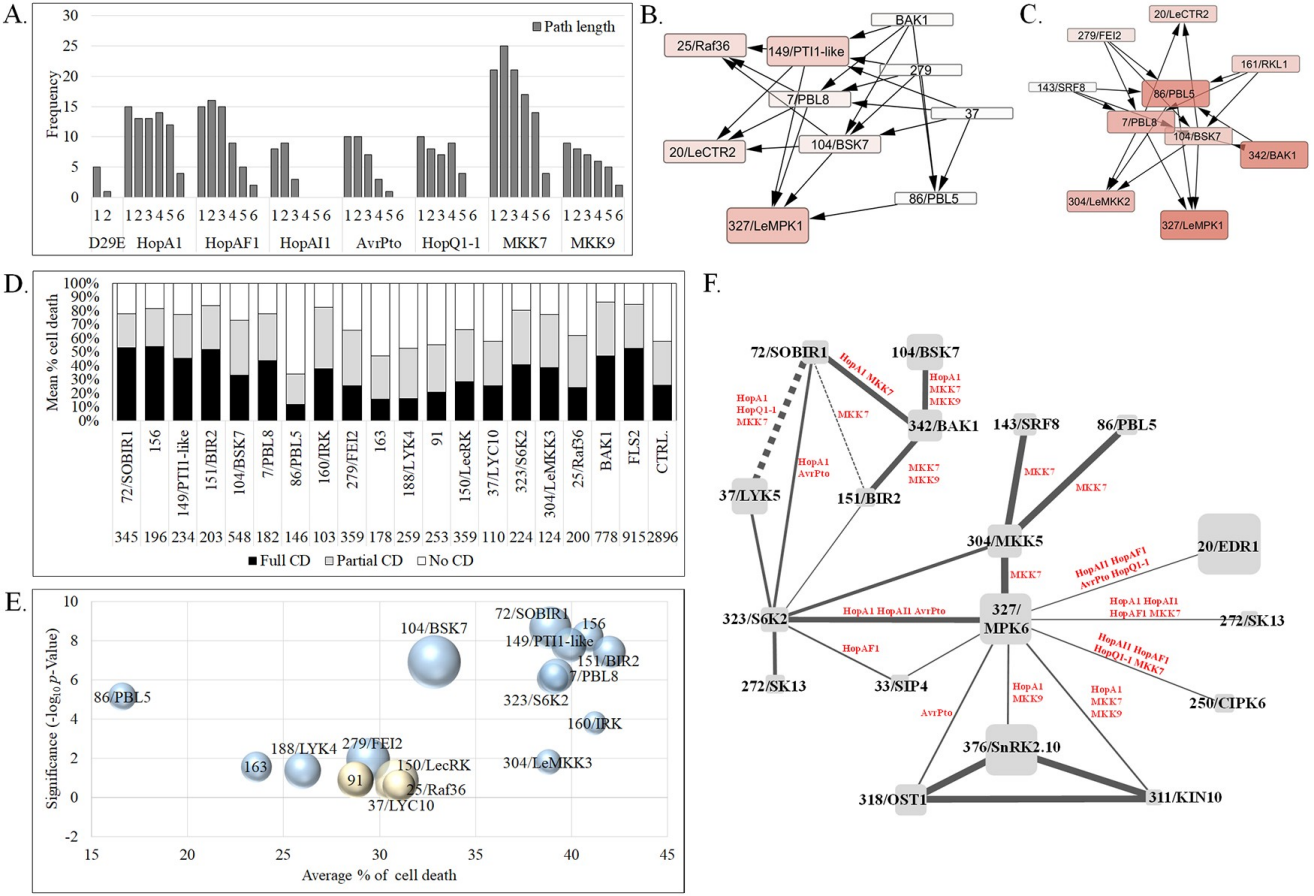
Topological parameters and predictive power of the signal-specific *KEI* networks. **A.** Comparison of the distribution of the shortest path length in the signal-specific networks D29E, D29E + HopA1, D29E + HopAF1, D29E + HopAI1, and D29E + AvrPto shown in 5C. Frequency of path length (of one to six nodes) was calculated using the NetworkAnalyzer [78] in Cytoscape 3.6.1. **B**–**C.** Sub-graphs of essential nodes in the defense signal-specific networks (B: cumulated HopA1, HopAI1, HopAF1, and AvrPto) and cell death networks (C: cumulated HopQ1-1, MKK7, and MKK9) (E) networks (shown in Fig 5C), computed using the topological scoring method of MCC. The MCC score is visualized in the node size and color (larger, darker color → higher score). MCC was computed using the cytoHubba app [79] and visualized using Cytoscape 3.6.1. **D**. Scoring of cell death in *Nicotiana benthamiana* leaves silenced for individual kinases and challenged with the nonhost pathogen *Pseudomonas fluorescens*, followed by the avirulent pathogen DC3000. Percentages of infiltrated areas showing various intensities of cell death are plotted for plants silenced for *KEIs* or with a control plasmid (EC1). Cell death was scored as absent (0%–25% death in the infiltrated area), partial (26%–75% cell death), or full (76%–100%). *N* shows the total number of replicates for each line tested. **E.** Bubble plot visualizing the cell death intensity and regulatory strength (−log_10_ of *p*-value) of *KEIs* tested in D. Blue bubbles show *KEIs* with statistically significant *p*-values; off-white bubble *KEIs* showed no statistical difference compared with the EC1 control. The size of bubbles is proportional with *N* shown in D. **F.** A weighted interaction network of *Arabidopsis* homologs of *KEIs* based on curated associations downloaded from STRING v10 [80]. Edges represent PPIs (continuous lines) or co-expression (interrupted lines). Edge weights are proportional with confidence scores calculated in STRING v10, scaled between zero and one, and indicating the likelihood that interactions are biologically meaningful, specific, and reproducible. Edge labels indicate the signal-specific networks in which connected KEIs are co-occurring. CD, cell death; *KEI*, *Kinase Effector Interactor*; MCC, Maximal Clique Centrality; PPI, protein–protein interaction.

Next, we generated networks representing the response of the minimal network under our eight experimental conditions, called stimuli-specific networks (SSNs), to reveal how the signaling network responded (Fig 5C). A comparison of the infection-response networks demonstrated that most of the D29E network is maintained across single-effector networks, with the exception of avirulence-inducing HopQ1-1. Addition of these single effectors affected the network topology, such that a larger number of *RLK* and *RLCK*s played a role. The networks doubled in diameter (the average length of shortest paths between all pairs of nodes) and had longer distance (shortest path index) between any two nodes, relative to the D29E network, indicative of activation of a more diverse and complex defense network (Fig 6A, S5A Fig). For example, *KEI104-BSK7* promoted immunity against strains containing HopA1, HopAF1, and AvrPto, but not the D29E strain. Interestingly, addition of virulence-promoting effectors (HopAI1, HopAF1, and AvrPto) activated ETS pathways, including cytosolic kinases *KEI339*, *KEI323/S6K2*, and *KEI318/SRK2C*. In the PCD networks, (HopQ1-1, MKK7, and MKK9), the signaling pathways were markedly distinct. While the MKK7 network is primarily comprised of *KEIs* that repress cell death, the MKK9 and HopQ1-1 networks were comprised of *KEIs* that promote cell death, densely populated with several *RLK* modules feeding into many cytosolic *KEIs*, and with most of the components functioning as negative regulators of the PCD; few nodes were shared. In the MKK7 and MKK9 networks, the diameter and path length indices were similar to HopQ1-1 (Fig 6A; S5A Fig). Signaling flow through SSNs converged onto a set of *KEIs* associated with the global control of transcription and translation, ion and nutrient homeostasis, and extracellular acidification. Examples include *KEI250/CIPK6* [40–42], *KEI33/CIPK11* [43], *KEI255/CIPK25* [44], *KEI323/S6K2* [45], and *KEI311/KIN10* [46].

To measure relative importance of the nodes in the SSNs we used maximum clique size algorithm (MCC) [47], which finds clusters of the largest size in a given network; sub-graphs of essential nodes were derived based on their MCC rank for the PTI, ETS (Fig 6B) and ETI, PCD (Fig 6C). Cytosolic kinases from the RLCK and MAPK-like families were preponderant essential nodes in both MCC-ranked graphs. Another parameter measuring centrality in networks is the betweenness centrality (BC) index, also regarded as a measure of the control potential of a node within a network [48]. Among all SSNs, the average BC indices were higher for MKKs and HopQ1-1 networks, indicating the importance of individual nodes on signaling outcome (S5B Fig). In contrast, the signaling networks associated with PTI and ETS (D29E, HopA1, HopAI1, HopAF1, and AvrPto) were smaller and had fewer high-control nodes (low-centrality nodes), implying decreased efficiency in signal transmission.

### Network-based predictions reveal novel immunity-associated kinases

To determine if the SSN networks could be used to predict the performance of genes in the defense response, we tested 18 *KEIs* for their role in basal immunity in *N*. *benthamiana*. In this assay, immunity is first induced by inoculation with a non-pathogen (*P*. *fluorescens*), followed by inoculation with the ETI-inducing *P*. *syringae* [49]. In the region where the inoculation areas overlap, little visible cell death develops, likely because of induced defense responses, limiting bacterial proliferation and secretion of the HopQ1-1 avirulence protein [50]. Most *KEI-*silenced lines had significantly increased cell death in the area infiltrated with both strains, indicating an impaired immune response as compared with the EC1 control and known immunity-promoting kinases (*BAK1* and *FLS*2) (Fig 6D; S1 Data**)**. Six of the eight highly MCC-ranked *KEIs*, including *KEI149/PTI1*-like, *KEI104/BSK7*, *KEI86/PBL5*, and *KEI7/PBL8*, had statistically significant phenotypes; others, including *KEI156*, *151/BIR2*, *160/IRK*, *323/S6K2*, *304/LeMKK3*, and the PTI-promoting *72/SOBIR1*, also exhibited significant differences in the cell death intensity compared with controls (Fig 6E). *KEI91* LRR RLK, which had no significant phenotypes in the ETS, ETI, or PCD phenotyping, showed control-level cell death. The known and curated protein–protein interactions (PPIs) for the *Arabidopsis* homologs of these *KEIs* were extracted from public databases and used to generate a network (Fig 6F), as described in the Supporting information (S1 Materials and Methods**—KEI signaling network analysis)**. The network has a PPI enrichment *p*-value of 3.1 × 10^−8^, indicating that most of these kinases are biologically connected among themselves. To further test our SSNs predictions, we overlapped the information from our eight orthogonal phenotyping assays over the PPI network. The nodes connected by 70% of the edges (17 out of 25) co-occurred in various SSNs, indicating they may also be functional partners. Notably, 65% of edges connecting the interacting and functionally related *KEIs* co-occurred in more than two SSNs. For example, the interacting pairs KEI327/MPK6 and KEI323/S6K2 were part of the HopA1, HopAI1, and AvrPto SSNs. Overall, these results indicate the predictive potential of the SSNs for mapping plant defense networks and their response to perturbations.

## Discussion

Plant immunity is generated as a result of numerous coordinated cellular processes. The study of inducible plant immunity requires approaches that reveal the organization and dynamics of the overall system and generate predictions on how molecular-level interventions can modify plant phenotypes. Building and characterizing biological networks, as a system-level approach to study plants, is starting to prove its effectiveness in predicting the function of cellular components and identifying biochemical and functional relationships among them [15, 51–54]. Here, we describe a network-driven integrative analysis of the plant immune system, which includes in vivo plant–pathogen interactomics and a comprehensive study of kinase targets and identification of signal-specific networks. Some of the findings revealed by our approach included (1) that some effectors may bind several tomato kinases and that a proportion of kinases can interact with multiple effectors, (2) defense-associated kinase networks contain both shared and specific nodes involved in basal immunity, ETS, ETI, and PCD, (3) effector-triggered kinase networks are larger and more complex compared with a basal-defense network; however, they have fewer nodes with high centrality than unperturbed networks, and (4) previously uncharacterized kinases are essential for promoting bacterial resistance in *N*. *benthamiana*. A comprehensive characterization of the kinases identified in this study can provide insights into the underlying molecular mechanisms of defense and on the sensitivity and response to perturbations of plant defense networks, and will help identify targets for genome editing in crops.

Our K-E screen predicts that interactions between plant proteins and pathogen effectors occur with a relatively low specificity when compared, for example, with receptor–ligand interactions. These observations confirm previous assumptions regarding effector promiscuity in target selection [13, 55, 56] and are supported by work demonstrating the functional interchangeability of *P*. *syringae* effectors [4]. By associating with multiple elements of a pathway, an effector may increase its chances to interfere successfully with the plant immune response. Furthermore, it may be evolutionarily beneficial for effectors to maintain the ability to interact with diverse partners to ensure functionality in new plant hosts with divergent immune signaling pathways [57–61]. On the other hand, HopM1 did not interact with any of the tested tomato kinases, suggesting a degree of target selectivity for some effectors. Indeed, target selectivity is further indicated by the fact that not all members of a kinase family interacted with the same effector. While this may be due to our experimental system, because effectors are rarely present individually and high expression of both kinase and effectors likely increased the chances for false positives, the effectors interacted with a mostly shared set of defense-associated kinases, suggesting the functional relevance of these interactions. Thus, while these interactions will have to be confirmed by additional methods, our findings indicate the effectiveness of using effector interactions as a starting point for genetic characterization.

Interestingly, several effectors interacted with both positive and negative modulators of immunity, demonstrating that interaction alone is not sufficient to predict the role of a host target in defense (HopA1 and PK3 or BAK1). During pathogenesis, effectors have additive or synergistic effects on promoting virulence in plants, and the impact of individual effectors on immunity is typically minor or nonsignificant [62]. The overall contribution of individual effectors is likely dependent on both the relative importance of individual host targets within the defense network and on the status of the network itself, as different sectors are inactivated by other effectors. In addition, essential kinases such as BAK1 often play dual roles, depending on the status of other regulatory kinases in the cell. For example, *AtBAK1* is essential for activation of PTI, but overaccumulation of AtBAK1 or loss of its negative regulator AtBIR1 can also activate immunity [63]. In biological networks, elimination of highly connected nodes (hubs) increases the diameter of the network [64] and has a deleterious effect on the characteristic path length and network integrity [65] compared with the removal of low-connectivity nodes. In this study, effectors appeared to neutralize hubs and nodes with high control potential in the network, thus having a detrimental effect on the structural integrity of the plant immune network. In addition, networks expanded in the presence of effectors, which may indicate plant deployment of new signaling sectors during purturbation.

Interestingly, several susceptibility-linked *KEIs* were identified across defense networks. These *KEI*s may act as negative immune regulators or could be recruited to subvert plant pathways for the benefit of the pathogen [66, 67]. Our results postulate that the composition and topology of plant signaling networks are determined by the plant’s ability to identify damage from effectors and activate compensatory pathways. Conversely, effector strategies to increase pathogen virulence consist in blocking/inactivating the sensor layers (*RLK/RLCK* modules) and recruiting kinases in the lower layers of the network for increasing pathogen fitness.

Comparison of the MKK7 and MKK9 networks suggests an antagonistic relationship between the pathways activated by these MAP2Ks, whereby activation of one may cause inhibition of the other. *MKK7* is a positive regulatory component of the immune response and systemic acquired resistance, operating via salicylic acid (SA) synthesis [68], while MKK9 positively regulates ET signaling through increasing ETHYLENE-INSENSITIVE3 (EIN3) receptor stability [69]. The complex functional relationship between SA and ET, comprising both synergistic [70, 71] and antagonistic [72] interactions, provides additional strength to this model.

Together, our network-centered approach has revealed the effect of individual effectors on signaling network topology and has facilitated the identification of novel immune kinases. However, several questions remain, including how effectors work together to modify the host immune network and if this information can be used to accurately predict the outcome of plant–pathogen interactions. A combination of systems biology approaches and genome editing has the potential to help address these questions and further the development of resistant plants for agricultural production.

## Materials and methods

### Bacterial strains

The coding region of the effector genes without the stop codon was cloned into the pENTR/SD/D-TOPO. The sequence for HopAI1 was amplified from *P*. *syringae* pv. *tomato* T1 using primers 5′-caccatgctcagtttaaagctgaacacccag and 5′-gcgagtccagggcggtggcatcag. All other effectors were obtained from *P*. *syringae* pv. *tomato* DC3000. Hrp promoter-driven effectors fused at the C terminus with the HA tag were generated in the destination vector pCPP5372 [73] using Gateway cloning. pCPP5372 carrying different effectors was mobilized into DC3000D29E, a derivative of DC3000D28E lacking HopAD1, by triparental mating using the helper plasmid pRK2013; *Trans*-conjugants were selected on KB medium with appropriate antibiotics. DC3000D28E::ShcM HopM1 has been described previously [74]. Bacteria were maintained on King’s B medium at 37 °C.

### Cloning and SLC

Cloning of the tomato *KEIs* and the SLC method were described previously [21]. To create clones for VIGS of orthologous kinases in *N*. *benthamiana*, tomato gene sequences were analyzed using bioinformatics tools available at solgenomics.net; the VIGS tool and the optimal gene fragment with the fewest off-targets were used to design primers. Gene fragments were amplified from *N*. *benthamiana* cDNA, cloned into the TOPO pER8 Donor vector using the manufacturer’s protocol, subcloned into the TRV2 expression vector, and transformed into *Agrobacterium* GV2260 for expression in planta [75]. Each interaction was tested in 4 to 16 independent assays, and the reconstituted luminescence was recorded at six time points. The decision to test over the minimum of four times was taken for the pairs showing significant levels of interaction when compared with the reference sets, while up to 16 assays (four biological replicates) were used for K-E pairs showing variability or low interaction levels. The interactions were corrected for multiple testing with a false discovery rate (FDR) of of 0.05. The analysis of SLCAs is described in S1 Materials and Methods.

### Viral-induced gene silencing, testing, and maintenance of KEI-silenced lines

The *KEI*-silenced lines were produced by syringe-infiltrating leaves of 2-week-old *N*. *benthamiana* plants with the TRV2-KEI *Agrobacterium* clones along with TRV1-containing *Agrobacterium* at a 1:1 ratio as described [75]. The EC1 and FLS2 constructs [49] served as controls and were included in each round of *KEI* line testing. *KEI* lines were grown (16 light, >50% humidity) in 6-inch-diameter pots for 3 weeks before testing. All functional assays were done using the third and fourth fully expanded leaves.

### Bacterial growth assays

Bacterial growth was tested in infiltrated leaves at 6 dpi. Each plant was tested once with each strain, and three plants were tested per round of *KEI*-silenced line production. Each *KEI*-silenced line was tested over a minimum of 3 and maximum of 18 trials alongside the controls *EC1*- and *FLS2*-silenced lines, resulting in between 9 and 56 biological replicates per *KEI–P*. *syringae* strain combination. The analysis of bacterial growth assays is described in S1 Materials and Methods.

### PCD induction

Two to three leaves of *KEI*-silenced lines were syringe infiltrated with *Agrobacterium* carrying the *MKK7*^*DD*^ or *MKK9*^*DD*^ as described previously [1]. The D29E + HopQ1-1 strain was applied by syringe inoculation at a level of 3 × 10^8^ CFU/mL. For both MAP2Ks and HopQ1-1 induction of PCD, the area of infiltration was marked, and the intensity of PCD was quantified over the 3 days after infiltration (dpi), as in [1]. The PCD was scored as 1 = 0%–25%, 2 = 26%–50%, 3 = 51%–75%, and 4 = 76%–100% of the infiltration area demonstrating necrosis. The data presented are using values at 1 dpi for HopQ1-1 and 2 dpi for MAP2K treatments. All three treatments were applied to the same leaf, and three plants were infiltrated per each round of VIGS. A minimum of three biological replicates were performed; 9 to 34 plants were tested per treatment. The analysis of PCD assays is described in S1 Materials and Methods.

### Phylogenetic and phenotypic analysis of KEIs

We used Clustal Omega tool [76] to align the sequences and iTOL [77] to build the phylogenetic tree of the 35 analyzed KEIs (Fig 4F and S2 Data). A sharable link is provided at https://itol.embl.de/tree/13018201144195851480695765#. We constructed a table of KEIs’ role in immune response using as rows the KEIs (clustered along the gene family structure using phylogenetic analysis) and as columns the classes of immune responses: PTI, ETS, ETI, MKK7ox, and MKK9ox. Each kinase was represented by an importance score, defined as the cumulative phenotype strength in each analyzed process:

$$IS(Ki,P)\;=\;\sum_{s\in P}-{log}_{10}\;\left(pVal(Ki,s)\right);\;P,\;immune\;reponse\;class;s,stress;Ki,kinase.$$

The heat map displays the importance scores of kinases versus immune processes (red: positive effect on immune response; blue: negative effect on immune response). The map shows a pattern of distinct RLKs having positive effect in PTI, ETS, and ETI (presumably by triggering immune responses), while cytosolic kinases have a negative effect on all immune response processes (possibly by contributing to pathogen growth and spread).

### Co-occurrence pattern analysis for signaling pathway inference

The network inference model uses the following matrices: (1) networks effect matrix: a matrix containing the decisions of phenotype testing on stress assays for each KEI; (2) phenotype effect matrix: a matrix containing the phenotype effect (positive or negative) of the stress assays for each KEI; and (3) co-occurrence matrix: a matrix containing the number of co-occurrences of pairs of kinases in treatments. In addition, it contains a set of structural constraints and rules for network structure inference.

The objective of our pathway inference method is to minimize the maximum co-occurrence pattern divergence for nodes included in the same pathway. We use a co-occurrence pattern similarity measure defined as $S(A,B)\;=\;(\frac{\left|A⋂B\right|}{\left|A\right|})x(\frac{\left|A⋂B\right|}{\left|B\right|})$ and pattern overlap measure $O_{A}(A,B)\;=\;(\frac{\left|A⋂B\right|}{\left|A\right|}).$

We developed a method to infer the signaling graph (the corresponding adjacency matrix) from the network effect matrix, co-occurrence matrix, and phenotype effect matrix subject to the structural constraints rules. The method consists of the following steps:

1. Classify KEIs according to localization and functional category in a hierarchy of four classes: {1. *RLK*, 2. *RLCK*, 3. *MAPK*, 4. *OCK*}. Structure the node hierarchy according to KEIs’ canonical signaling pathways architecture (RLK → RLCK → MAP3Ks → MKKs → MAPK → OCK). Add known edges and other structural constraints (i.e., nodes that do not interact) to the network.
2. Compute network effect matrix, phenotype effect matrix, and co-occurrence matrices.
3. Construct the network using a method consisting of two stages:
  - Network grow: Add edges to signaling network using co-occurrence information. Only interlayer edges are added; KEIs are in adjacent layers and co-occur in at least T_1_ assays, and the similarity of phenotype patterns is greater than T_2_.
  - Network trim: Analyze network motifs to trim the structure of the network. Rearrange the motif of connected intra-layer nodes (module) so as to push network nodes with the largest co-occurrence score toward the center (MAPK cascade layer) and to push nodes with smallest co-occurrence score toward the in and out of the network.
  - Add intra-layer edges: KEIs are from the same layer, and the similarity of co-occurrence pattern is at least T_3_.
  - Remove edge: After motif rearrangement, remove inter-layer edges between nodes that are not adjacent. Remove the intra-layer edges between nodes at the same level in the module.
4. Network complete: Add edges across layers—skip edges (weak edges, dotted) to account for missing pathways and components if (1) KEIs cannot be connected with interlayer or intra-layer edges due to low co-occurrence or (2) KEIs have a very high similarity of co-occurrence patterns with a node from a skip layer.

T_1_, T_2_, and T_3_ are control parameters with geometric rate convergence to equilibrium values.

## Supporting information

S1 Fig Processing and visualization of protein interaction data from the high-throughput SLCAs. **A.** The luminescence signals of technical replicates located in the same 96-well plate show low variability. Measurements of correlation coefficients on data sets from biological replicates (T_1_ to T_4_) show high repeatability: T_1_–T_2_ (*r* = 0.98), T_3_–T_4_ (*r* = 0.962), T_1_–T_4_ (*r* = 0.962), and T_2_–T_3_ (*r* = 0.979). Only T_1_–T_2_ and T_3_–T_4_ are shown in the scatterplots. **B.** Scatterplots of normalized signals of controls from the SLCAs. Shown are scatterplots of Luciferase versus Pto–AvrPto, Luciferase versus Pto–AvrPto^I96A^, and Luciferase versus Background normalized signals. Correlation coefficients: corr = 0.44 (Luciferase versus Pto–AvrPto), corr = 0.55 (Luciferase versus Pto–AvrPto^I96A^) and corr = −0.053 (Luciferase versus Background). **C.** Scatterplot of K-E probes versus controls (normalized signals). There is no correlation between K-E probe signals and the positive or negative control, indicating the lack of a measuring bias in our protocol (note: control signals are common for each 96-well plate tested, with 20 K-E probes per plate). **D.** Multiple regression of Pto–AvrPto^I96A^ versus Pto–AvrPto and Luciferase signals has *R*^2^ = 0.887, adjusted *R*^2^ = 0.886, and regression coefficients 7.17 × 10^−3^ (Luciferase) and 3.06 × 10^−1^ (Pto–AvrPto). **E.** The distribution of normalized signals for controls and K-E probes. corr, correlation; K-E, kinase–effector; SLCA, split-luciferse complementation assay.
(TIF)

S2 Fig Characteristics of pairwise K-E interactions measured in the SLCAs. **A.** A scatter chart showing the distribution of values for the interaction strength (fold change versus control) for all K-E interactions tested. **B.** The distribution of *KEIs* across protein kinase families. Percentages represent the number of kinases interacting with effectors from each group. K-E, kinase–effector; SLCA, split luciferase complementation assay.
(TIF)

S3 Fig Characterization of *KEIs’* roles in plant innate immunity. **A.** Measurement of transcript accumulation following viral-induced gene silencing of *KEI* homologs in *Nicotiana benthamiana* by quantitative RT-PCR, in control (*EC1*)-silenced plants (black columns), and *KEI*-silenced plants (white columns). Asterisks denote significance (*p* < 0.01). **B.** Bacterial growth assays in control (*EC1*) or *KEI*-silenced *N*. *benthamiana* leaves. After silencing, plants were challenged with five *Pseudomonas syringae* mutant strains: D29E, D29E + HopA1 (A1), D29E + HopAI1 (AI1), D29E + HopAF1 (AF1), and D29E + AvrPto (AvrPto). Bacterial growth was measured as described in Materials and methods, and values were plotted as CFU per area of sampled tissue. **C.** The *p*-value of all *KEIs* tested as described in (B). The x-axis crosses the y-axis at *p* = 0.01. The *KEIs* are grouped according to their structural homology. **D.** Pie chart showing percentages of *KEIs* out of the total tested with statistically significant phenotypes in bacterial growth assays for each *P*. *syringae* strain tested in (B). CFU, colony forming unit; *KEI*, *Kinase Effector Interactor*; RT-PCR, quantitative real-time PCR.
(TIF)

S4 Fig Characterization of *KEIs* roles in PCD. **A.** Histogram showing the intensity of cell death measured in control (*EC1*) or *KEI*-silenced *Nicotiana benthamiana* leaves following challenge with a bacterial strain triggering effector-induced immunity, *Pseudomonas syringae* D29E +HopQ1-1, or after overexpression of the MAP2Ks, MKK7 or MKK9. Cell death intensity was assessed using the scoring scale shown in the inset and as described in materials and methods. **B.** Histogram showing the regulatory strength (log_10_ of p-value) of all *KEIs* tested for cell death-associated phenotypes (inoculation with D29E +HopQ1-1, and overexpression of MKK7 or MKK9). The X-axis crosses the Y-axis at p-value = 0.01, and the position of the 0.01 and 0.05 p-values are indicated with red lines. The *KEIs* are grouped according to their structural homology in this particular order from left to right: *RLKs/RLCKs*, *MAPKs*, and Other kinases. *KEI*, *Kinase Effector Interactor*; MAPK, MAP kinase; PCD, programmed cell death; RLCK, receptor-like cytosolic kinase; RLK, receptor-like kinase.
(TIF)

S5 Fig Topological parameters of signal-specific networks. **A.** Topological and statistical parameters of the signal-specific networks shown in Fig 5C. **B.** Visualization of average BC of the signal-specific networks shown in Fig 5C. All parameters were calculated using the NetworkAnalyzer in Cytoscape v. 3.6.1. BC, betweenness centrality.
(TIF)

S1 Data Primary data for all phenotypic assays performed in this study: Bacterial growth assay, ETI and MKK overexpression cell death measurements, and PTI cell death suppression assays. ETI, effector-triggered immunity; MKK, MAP kinase kinase; PTI, PAMP-triggered immunity.
(XLSX)

S2 Data Phylogeny analysis data. (TXT)

S3 Data MATLAB code for SLC data analysis. SLC, split-luciferase complementation.
(PDF)

S4 Data Alignment and tree files for the phylogenetic tree in Fig 4F. (RAR)

S1 Table A list of *KEIs* and interacting effector(s). The list was used to generate the K-E network from Fig 2A. K-E, kinase–effector; *KEI*, *Kinase Effector Interactor*.
(DOCX)

S2 Table A list of the focus *KEIs* including the number, https://solgenomics.net/ ID, the top BLAST hit in the *Arabidopsis* genome, known symbol, kinase structural class in PlantsP database (https://www.hsls.pitt.edu/obrc/index.php?page=URL1100616073), and their bacterial effector interactors. BLAST, Basic Local Alignment Search Tool; *KEI*, *Kinase Effector Interactor*.
(DOCX)

S3 Table VIGS amplicons used in this study. The table lists the gene lab ID, the sequences of the PCR oligonucleotides used for amplification, the expected size of the PCR amplicon, the gene ID in tomato and *Nicotiana benthamiana*, the number of targeted genes, and the length of overlap between the target and PCR amplicon. The last two columns list potential off-target genes as well as the maximum length of the off-target regions. The PCR oligonucleotides were tested using the SGN VIGS tool at http://solgenomics.net/tools/vigs. SGN, SolGenomics Network; VIGS, virus-induced gene silencing.
(DOCX)

S4 Table The table used to generate Fig 2C. (XLSX)

S5 Table The table used to generate Fig 2D and S2B Fig. (XLSX)

S6 Table The table used to generate Fig 4E. (XLSX)

S7 Table The table used to generate Fig 6F. (XLSX)

S8 Table The table used to generate S1 Fig. (XLSX)

S9 Table The table used to generate S2A Fig. (XLSX)

S10 Table The table used to generate S3B Fig. (XLSX)

S11 Table The table used to generate S3C Fig. (XLSX)

S12 Table The table used to generate S4A Fig. (XLSX)

S13 Table The table used to generate S4B Fig. (XLSX)

S14 Table The table used to generate S5B Fig. (XLSX)

S15 Table The table used to generate Fig 6A. (XLSX)

S16 Table The table used to generate Fig 6D. (XLSX)

S17 Table The table used to generate Fig 6E. (XLSX)

S1 Materials and Methods The supplemental methods file contains details on (1) SLCA data analysis, (2) statistical testing of bacterial growth and PCD assays for VIGS-silenced KEIs, (3) correlation of immune responses for KEIs with significant stress phenotype, (4) co-occurrence pattern analysis for immune pathways inference, and (5) KEI signaling network analysis. KEI, Kinase Effector Interactor; PCD, programmed cell death; SLCA, split luciferase complementation assay; VIGS, virus-induced gene silencing.
(DOCX)

## Abbreviations

BAK1: BRI1-ASSOCIATED RECEPTOR KINASE1
BC: betweenness centrality
EFR: EF-TU RECEPTOR
EIN3: ETHYLENE-INSENSITIVE3
ET: ethylene
ETI: effector-triggered immunity
ETS: effector-triggered susceptibility
FDR: false discovery rate
FLS2: FLAGELLIN-SENSING2
GSK3: glycogen synthase kinase3
K-E: kinase–effector
KEI: *Kinase Effector Interactor*
LRR: leucine-rich repeats
MAP: mitogen-activated protein
MAPK: mitogen-activated protein kinase
MAP2K: MAP kinase kinase
MAP3K: MAP kinase kinase kinase
MCC: maximum clique size algorithm
MIN7: HopM1 interactor 7
MTN1: methylthioadenosine nucleosidase 1
MTN2: methylthioadenosine nucleosidase 2
PAMP: pathogen-associated molecular pattern
PCD: programmed cell death
PPI: protein–protein interaction
Prf: Pseudomonas resistance and fenthion sensitivity
PRR: pattern recognition receptor
Pst: *Pseudomonas syringae* pv. *tomato*
PTI: PAMP-triggered immunity
RLCK: receptor-like cytosolic kinase
RLK: receptor-like kinase
SA: salicylic acid
SLC: split luciferase complementation
SLCA: split luciferase complementation assay
SSN: stimuli-specific network
S6K: ribosomal protein S6 kinase
VIGS: virus-induced gene silencing
