# Supplementary figures and images for: Integrative network-centric approach reveals signaling pathways associated with plant resistance and susceptibility to Pseudomonas syringae
Source: PLoS Biol. 2018 Dec 12;16(12):e2005956. doi: 10.1371/journal.pbio.2005956 (PMC6322785; doi:10.1371/journal.pbio.2005956)

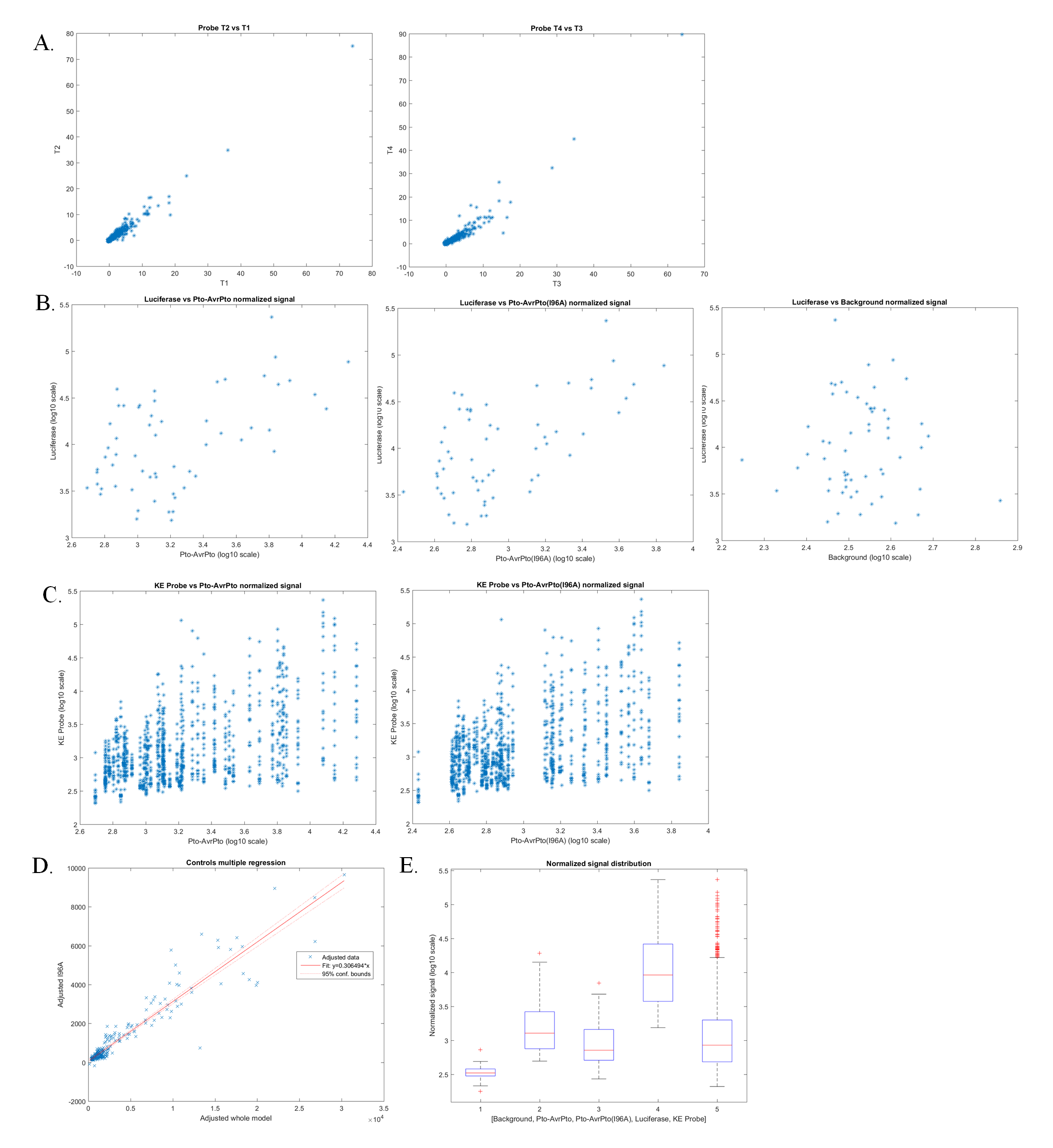

Supplement: S1 Fig — A. The luminescence signals of technical replicates located in the same 96-well plate show low variability. Measurements of correlation coefficients on data sets from biological replicates (T1 to T4) show high repeatability: T1–T2 (r = 0.98), T3–T4 (r = 0.962), T1–T4 (r = 0.962), and T2–T3 (r = 0.979). Only T1–T2 and T3–T4 are shown in the scatterplots. B. Scatterplots of normalized signals of controls from the SLCAs. Shown are scatterplots of Luciferase versus Pto–AvrPto, Luciferase versus Pto–AvrPtoI96A, and Luciferase versus Background normalized signals. Correlation coefficients: corr = 0.44 (Luciferase versus Pto–AvrPto), corr = 0.55 (Luciferase versus Pto–AvrPtoI96A) and corr = −0.053 (Luciferase versus Background). C. Scatterplot of K-E probes versus controls (normalized signals). There is no correlation between K-E probe signals and the positive or negative control, indicating the lack of a measuring bias in our protocol (note: control signals are common for each 96-well plate tested, with 20 K-E probes per plate). D. Multiple regression of Pto–AvrPtoI96A versus Pto–AvrPto and Luciferase signals has R2 = 0.887, adjusted R2 = 0.886, and regression coefficients 7.17 × 10^−3^ (Luciferase) and 3.06 × 10^−1^ (Pto–AvrPto). E. The distribution of normalized signals for controls and K-E probes. corr, correlation; K-E, kinase–effector; SLCA, split-luciferse complementation assay. (TIF) [file pbio.2005956.s001.tif]

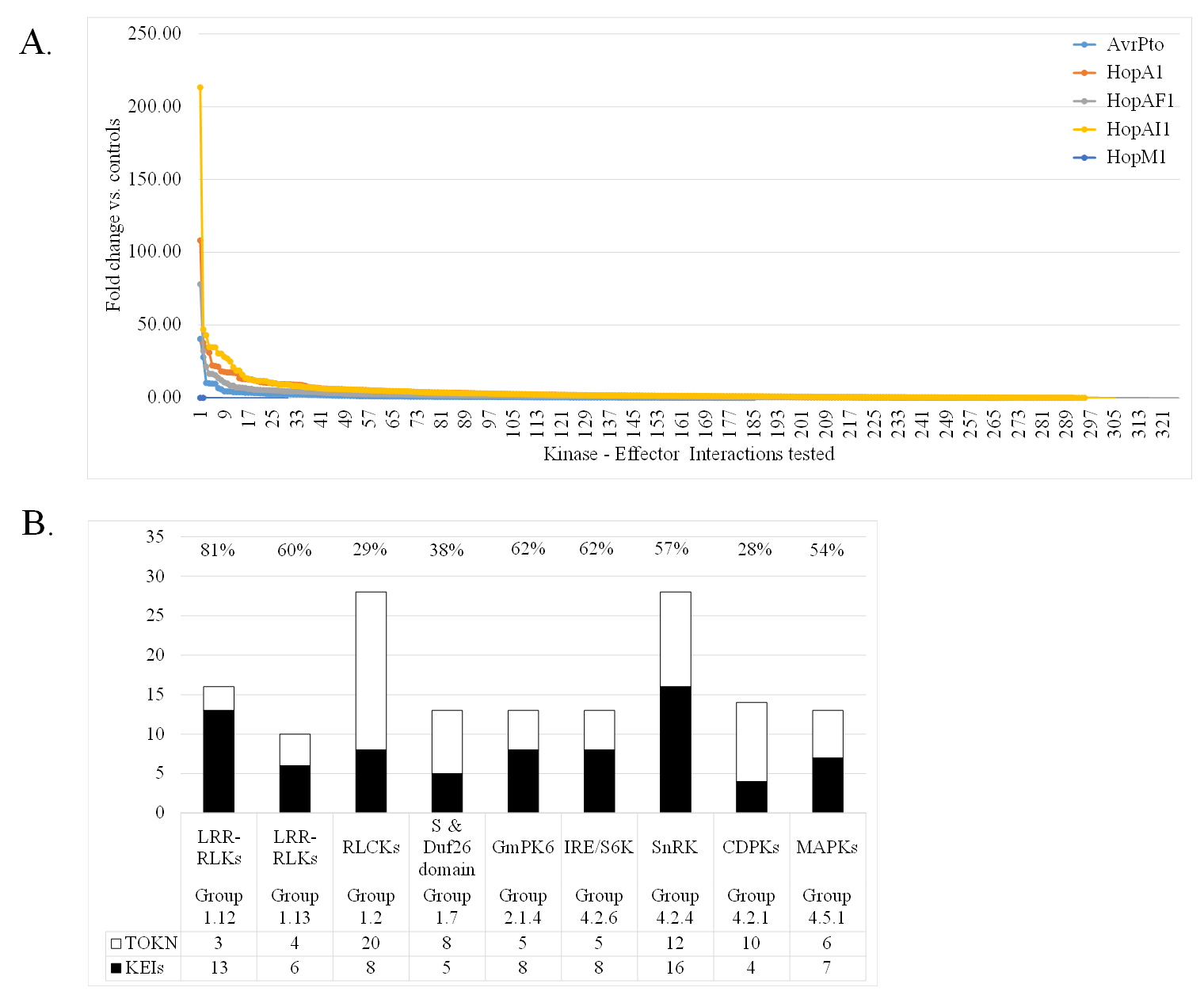

Supplement: S2 Fig — A. A scatter chart showing the distribution of values for the interaction strength (fold change versus control) for all K-E interactions tested. B. The distribution of KEIs across protein kinase families. Percentages represent the number of kinases interacting with effectors from each group. K-E, kinase–effector; SLCA, split luciferase complementation assay. (TIF) [file pbio.2005956.s002.tif]

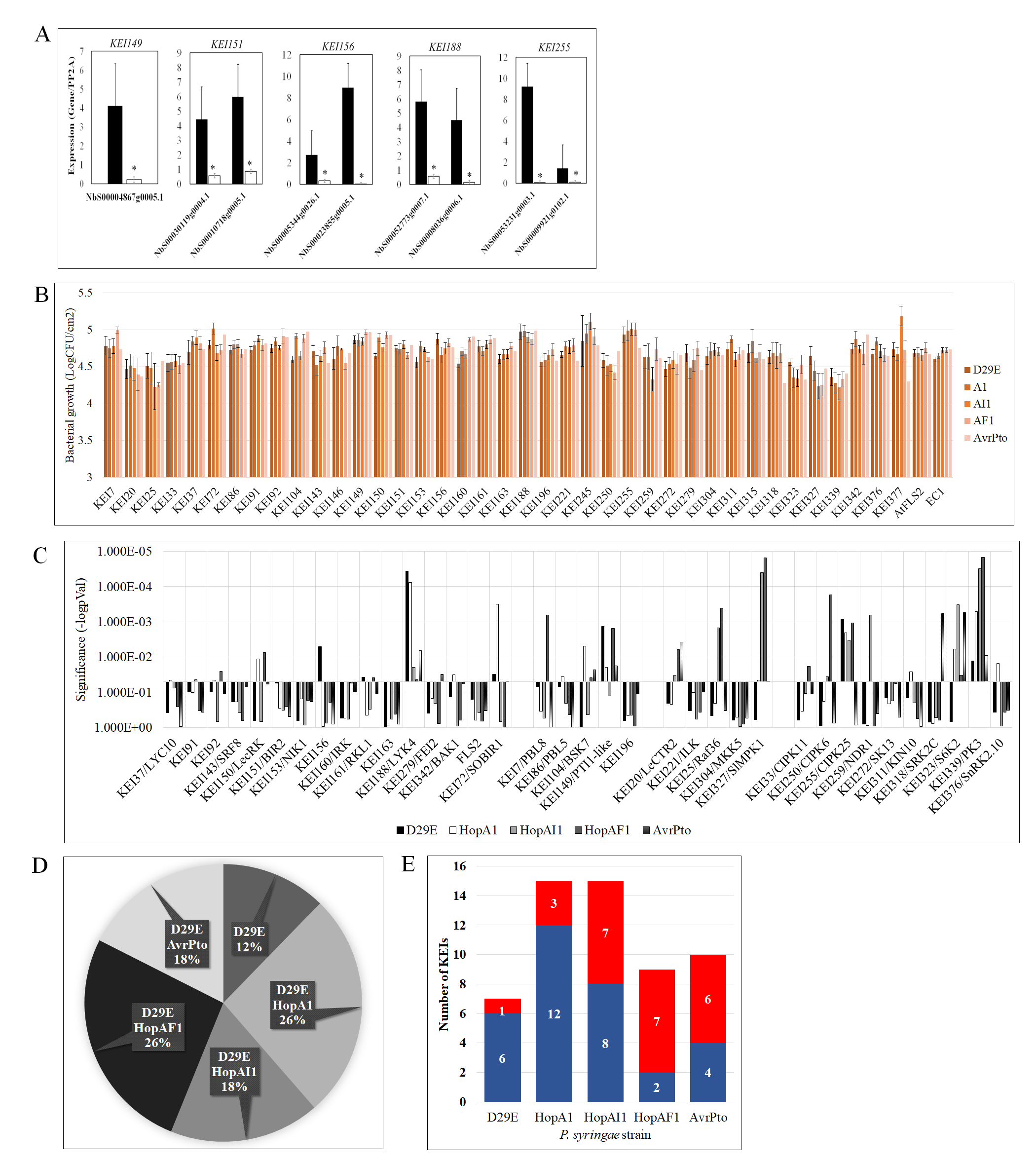

Supplement: S3 Fig — A. Measurement of transcript accumulation following viral-induced gene silencing of KEI homologs in Nicotiana benthamiana by quantitative RT-PCR, in control (EC1)-silenced plants (black columns), and KEI-silenced plants (white columns). Asterisks denote significance (p < 0.01). B. Bacterial growth assays in control (EC1) or KEI-silenced N. benthamiana leaves. After silencing, plants were challenged with five Pseudomonas syringae mutant strains: D29E, D29E + HopA1 (A1), D29E + HopAI1 (AI1), D29E + HopAF1 (AF1), and D29E + AvrPto (AvrPto). Bacterial growth was measured as described in Materials and methods, and values were plotted as CFU per area of sampled tissue. C. The p-value of all KEIs tested as described in (B). The x-axis crosses the y-axis at p = 0.01. The KEIs are grouped according to their structural homology. D. Pie chart showing percentages of KEIs out of the total tested with statistically significant phenotypes in bacterial growth assays for each P. syringae strain tested in (B). CFU, colony forming unit; KEI, Kinase Effector Interactor; RT-PCR, quantitative real-time PCR. (TIF) [file pbio.2005956.s003.tif]

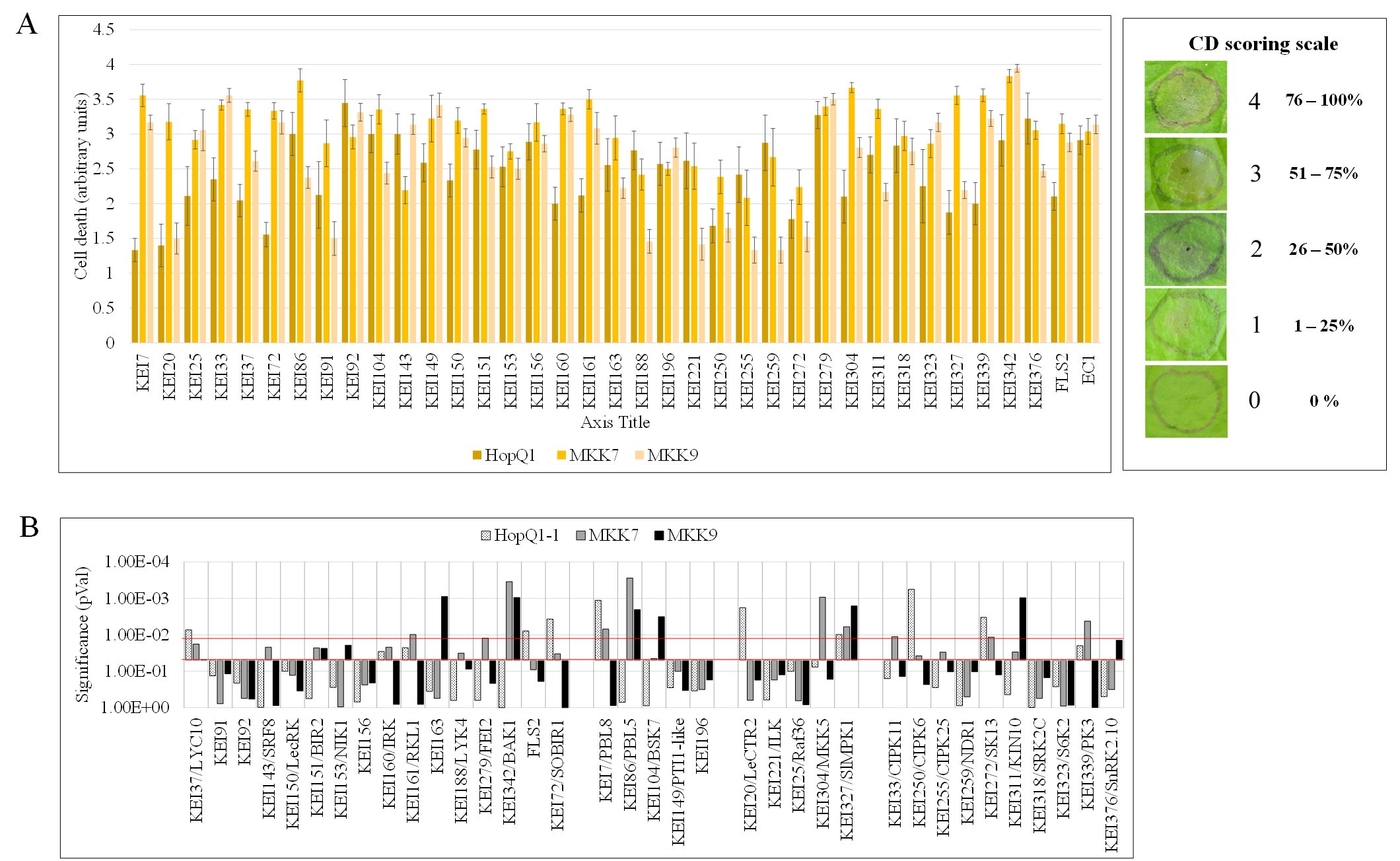

Supplement: S4 Fig — A. Histogram showing the intensity of cell death measured in control (EC1) or KEI-silenced Nicotiana benthamiana leaves following challenge with a bacterial strain triggering effector-induced immunity, Pseudomonas syringae D29E +HopQ1-1, or after overexpression of the MAP2Ks, MKK7 or MKK9. Cell death intensity was assessed using the scoring scale shown in the inset and as described in materials and methods. B. Histogram showing the regulatory strength (log10 of p-value) of all KEIs tested for cell death-associated phenotypes (inoculation with D29E +HopQ1-1, and overexpression of MKK7 or MKK9). The X-axis crosses the Y-axis at p-value = 0.01, and the position of the 0.01 and 0.05 p-values are indicated with red lines. The KEIs are grouped according to their structural homology in this particular order from left to right: RLKs/RLCKs, MAPKs, and Other kinases. KEI, Kinase Effector Interactor; MAPK, MAP kinase; PCD, programmed cell death; RLCK, receptor-like cytosolic kinase; RLK, receptor-like kinase. (TIF) [file pbio.2005956.s004.tif]

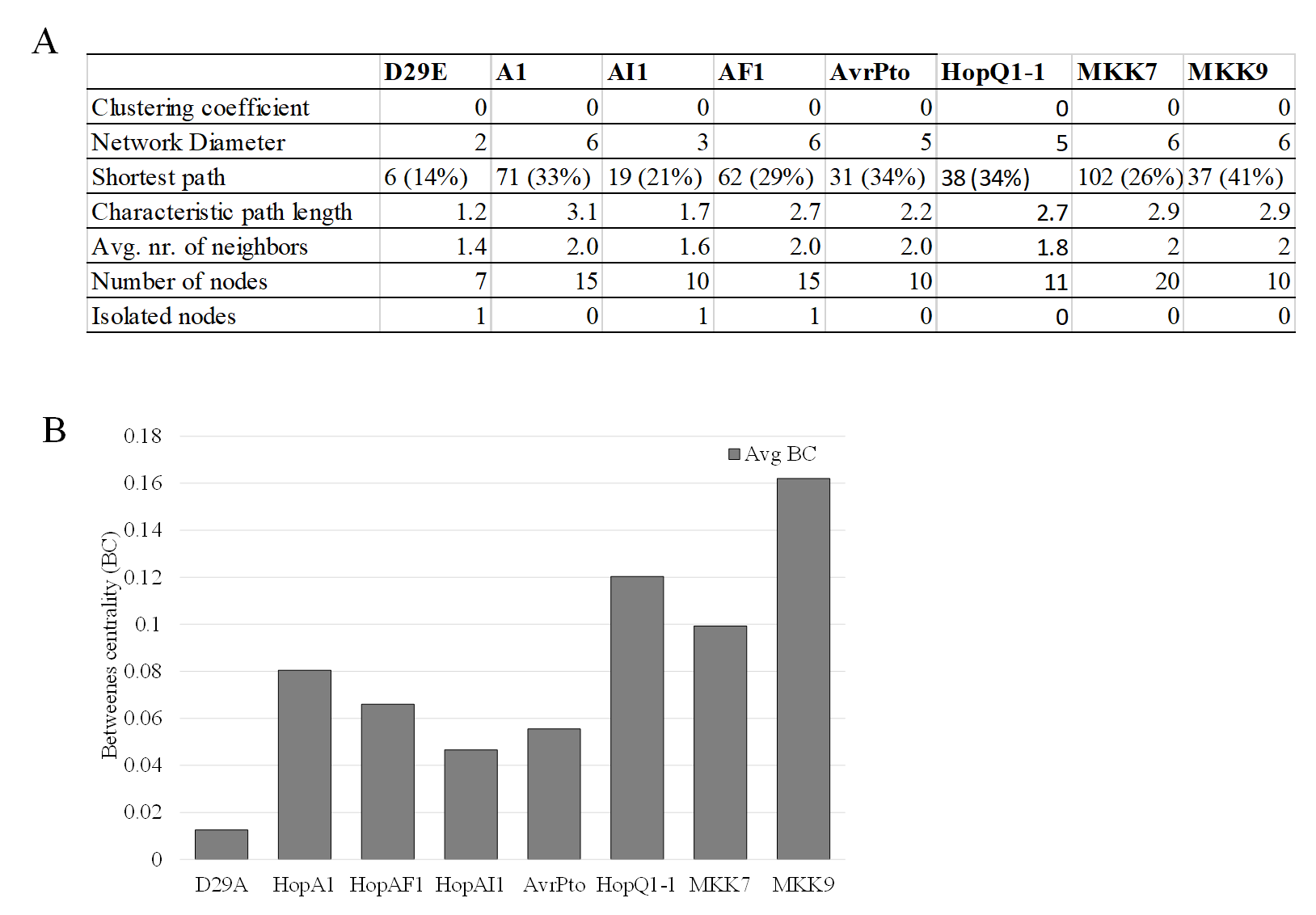

Supplement: S5 Fig — A. Topological and statistical parameters of the signal-specific networks shown in Fig 5C. B. Visualization of average BC of the signal-specific networks shown in Fig 5C. All parameters were calculated using the NetworkAnalyzer in Cytoscape v. 3.6.1. BC, betweenness centrality. (TIF) [file pbio.2005956.s005.tif]
