# Supplementary material for: Integrative network-centric approach reveals signaling pathways associated with plant resistance and susceptibility to Pseudomonas syringae
Source: PLoS Biol. 2018 Dec 12;16(12):e2005956. doi: 10.1371/journal.pbio.2005956 (PMC6322785; doi:10.1371/journal.pbio.2005956)
Supplement: S3 Data — SLC, split-luciferase complementation. (PDF) [file pbio.2005956.s008.pdf]

```
%%%%%%%%%%%%%%%%%%%%%%%%%%%
clear all;
close all;
format long e

%%%%%%%%%%%%%%%%%%%%%%%%%%
%%CONSTANTS
%%%%%%%%%%%%%%%%%%%%%%%%%%%
DATA_SET=2;
PRINT=1;

%% Params
%%%%%%%%%%%%%%%%%%%%
k_ctrl=0.5;

%%%%%%%%%%%%%%%%%%%%%%%%%%%%%%%%%%%%%%
%% Load data
%%%%%%%%%%%%%%%%%%%%%%%%%%%%%%%%%%%%%%
%% Read pilot data

[DIR,str_cell , set1, set2]=load_SLCA(DATA_SET);
%%%%%%%%%%%%%%%%%%%%%%%%%%%%%%%%%%
ssize=size(str_cell,2);
colSize=20;
repNum=4; %% Number of replicates per sample
ctrlNum=4; %% Number of Controls per plate: Bak, AvrPto, I96A, Luc

dist_matr=zeros(ctrlNum, ssize,colSize);
pos_ctrl=zeros(ctrlNum,ssize,colSize);
pos_matr=zeros(ssize,colSize);

d_matr=zeros(ctrlNum,ssize,colSize,repNum);
c_matr=zeros(ctrlNum,ssize,colSize,repNum);
c_matrn=zeros(ctrlNum,ssize,colSize,repNum);


%% Dataset: [effectors, breplicate/plate_index, time, tokn/ctrl, treplicate]
slca_matr=cell(ssize, 389); %% indexed by plate instead of effector (5)
ctrl_matr=cell(ssize, ctrlNum);
tokn_ind_matr=zeros(ssize,colSize);
eff_ind_matr=set1(cell2mat(set2)'*ones(1,colSize));
plateID=cell(ssize, colSize);
HVal=zeros(ssize,colSize);
pVal=zeros(ssize,colSize);
HVal_n=zeros(ssize,colSize);
pVal_n=zeros(ssize,colSize);
HVal_np=zeros(ssize,colSize);
pVal_np=zeros(ssize,colSize);
qVal=zeros(ssize,colSize);
I_Val=zeros(ssize,colSize);
I96_std_Val=zeros(ssize,colSize);
I96_m_Val=zeros(ssize,colSize);
std_Val=zeros(ssize,colSize);
m_Val=zeros(ssize,colSize);

str_prot=str_cell{1,1};
```

```
[matr_ctrl, matr_txt] = xlsread(strcat (DIR, str_prot),'Sheet1' , 'B3:E6' );

%%%%%%%%%%%%%%%%%%%%%%%%%%%
%% MAIN LOOOP
%%%%%%%%%%%%%%%%%%%%%%%%%%%

input('Main Loop')
%% Start Loop for protein strings
for ii=1:size(str_cell,2)

str_prot=str_cell{1,ii};
%%input('Processing string');
disp(str_prot);

%% Structure of the data matrix information: TOKN indices
matr_ind = xlsread(strcat (DIR, str_prot),'Sheet1' , 'B3:M10' );
tokn_ind_matr(ii,:)=[matr_ind(5:8,1)', matr_ind(:,5)', matr_ind(:,9)'];

%% Six time points

for jj=1:6
str_range=strcat('B',num2str(13+(jj-1)*10),':M', num2str(colSize+(jj-1)*10));
slca_probe = xlsread(strcat (DIR, str_prot),'Sheet1' , str_range );
data_m=[slca_probe(5:8,1:repNum); slca_probe(:,5:8); slca_probe(:,9:12) ] ;

set1(set2{ii})

%% SLCA datapoints
for(kk=1:size(tokn_ind_matr,2))
    if(tokn_ind_matr(ii,kk)<400 && tokn_ind_matr(ii,kk) )
    slca_matr{ii, tokn_ind_matr(ii,kk)}(jj,1:repNum)= data_m(kk,1:repNum);
    end
    plateID(ii,kk)=str_cell(ii);
end
%% CTRL datapoints
for(kk=1:ctrlNum)
ctrl_matr{ ii , kk }(jj,1:repNum)= slca_probe(kk,1:repNum);
end

end %%jj


for(kk=1:size(tokn_ind_matr,2))

%% CTRL distances
c_matr( 1, ii, kk, : )= sign(sum (ctrl_matr{ ii,3}(:,:) - ctrl_matr{ ii,1}(:,:))) .* sqrt↙
(sum ((ctrl_matr{ ii,3}(:,:) - ctrl_matr{ ii,1}(:,:)).^2));  %% I96A
c_matr( 2, ii, kk, : )= sign(sum (ctrl_matr{ ii,2}(:,:) - ctrl_matr{ ii,1}(:,:))) .* sqrt↙
(sum ((ctrl_matr{ ii,2}(:,:) - ctrl_matr{ ii,1}(:,:)).^2));  %% AvrPTO
c_matr( 3, ii, kk, : )= sign(sum (ctrl_matr{ ii,4}(:,:) - ctrl_matr{ ii,1}(:,:))) .* sqrt↙
(sum ((ctrl_matr{ ii,4}(:,:) - ctrl_matr{ ii,1}(:,:)).^2));  %% LUC

c_matrn( 1 , ii, kk, : ) = c_matr( 1 , ii, kk, : )./mean(c_matr( 3 , ii, kk, : ),4);
c_matrn( 2 , ii, kk, : ) = c_matr( 2 , ii, kk, : )./mean(c_matr( 3 , ii, kk, : ),4);
c_matrn( 3 , ii, kk, : ) = c_matr( 3 , ii, kk, : )./mean(c_matr( 3 , ii, kk, : ),4);
```

```
%% CTRL positions
pos_ctrl( 1, ii, kk ) = sqrt(sum (mean(ctrl_matr{ ii,1}(:,:)).^2));
pos_ctrl( 2, ii, kk ) = sqrt(sum (mean(ctrl_matr{ ii,2}(:,:) ).^2));
pos_ctrl( 3, ii, kk ) = sqrt(sum (mean(ctrl_matr{ ii,3}(:,:) ).^2));
pos_ctrl( 4, ii, kk ) = sqrt(sum (mean(ctrl_matr{ ii,4}(:,:) ).^2));


if(tokn_ind_matr(ii,kk)<400 && tokn_ind_matr(ii,kk) )
%%compute distances
dist_matr( 1 , ii, kk )= sign(sum (mean(slca_matr{ ii,tokn_ind_matr(ii,kk)}(:,:)') - mean↙
(ctrl_matr{ ii,1}(:,:)'))) * sqrt(sum ((mean(slca_matr{ ii,tokn_ind_matr(ii,kk)}(:,:)') -↙
mean(ctrl_matr{ ii,1}(:,:)')).^2));
dist_matr( 2 , ii, kk )= sign(sum (mean(slca_matr{ ii,tokn_ind_matr(ii,kk)}(:,:)') - mean↙
(ctrl_matr{ ii,2}(:,:)'))) * sqrt(sum (mean(slca_matr{ ii,tokn_ind_matr(ii,kk)}(:,:)') -↙
mean(ctrl_matr{ ii,2}(:,:)')).^2);
dist_matr( 3 , ii, kk )= sign(sum (mean(slca_matr{ ii,tokn_ind_matr(ii,kk)}(:,:)') - mean↙
(ctrl_matr{ ii,3}(:,:)'))) * sqrt(sum (mean(slca_matr{ ii,tokn_ind_matr(ii,kk)}(:,:)') -↙
mean(ctrl_matr{ ii,3}(:,:)')).^2);
dist_matr( 4 , ii, kk )= sign(sum (mean(slca_matr{ ii,tokn_ind_matr(ii,kk)}(:,:)') - mean↙
(ctrl_matr{ ii,4}(:,:)'))) * sqrt(sum (mean(slca_matr{ ii,tokn_ind_matr(ii,kk)}(:,:)') -↙
mean(ctrl_matr{ ii,4}(:,:)')).^2);

%%%%
d_matr( 1 , ii, kk, : )=  sign(sum (slca_matr{ ii,tokn_ind_matr(ii,kk) }(:,:) - ctrl_matr↙
{ ii,1}(:,:))).* sqrt(sum ((slca_matr{ ii,tokn_ind_matr(ii,kk) }(:,:) - ctrl_matr{ ii,1}↙
(:,:)).^2));
d_matr( 2 , ii, kk, : )= sign(sum (slca_matr{ ii,tokn_ind_matr(ii,kk) }(:,:) - ctrl_matr{↙
ii,2}(:,:))).* sqrt(sum ((slca_matr{ ii,tokn_ind_matr(ii,kk) }(:,:) - ctrl_matr{ ii,2}↙
(:,:)).^2));
d_matr( 3 , ii, kk, : )= sign(sum (slca_matr{ ii,tokn_ind_matr(ii,kk) }(:,:) - ctrl_matr{↙
ii,3}(:,:))).* sqrt(sum ((slca_matr{ ii,tokn_ind_matr(ii,kk) }(:,:) - ctrl_matr{ ii,3}↙
(:,:)).^2));
d_matr( 4 , ii, kk, : )= sign(sum (slca_matr{ ii,tokn_ind_matr(ii,kk) }(:,:) - ctrl_matr{↙
ii,4}(:,:))).* sqrt(sum ((slca_matr{ ii,tokn_ind_matr(ii,kk) }(:,:) - ctrl_matr{ ii,4}↙
(:,:)).^2));


%% average positions (norm)
pos_matr( ii, kk )= sqrt(sum ((mean(slca_matr{ ii,tokn_ind_matr(ii,kk)}(:,:)')).^2));


end %% if
end

end %%ii

%% Regression model to predict the inhibitory level from the controls
%% Normalized, pooled CTRL
v1=c_matr(1, :, 1, :);
v2=c_matr(2, :, 1, :);
v3=c_matr(3, :, 1, :);
tbl=table(v3(:), v2(:), v1(:), 'VariableNames',{'Luc','AvrPto','I96A'});
lm = fitlm(tbl,'I96A~AvrPto+Luc')
figure()
plot(lm);
```

```
if (PRINT)
title('Controls multiple regression')
print ('-dtiff', strcat(DIR, 'res/Controls_LM'))
end
%% table
str=strcat( DIR , 'res/Controls_LM.xlsx');
writetable(tbl, str);

input('Controls linear model regression')


%% plotting the curves
figure()
hold on
for (ii=1:ssize)
    plot(mean(ctrl_matr{ii,1}(:,:)'))

end
title('N-ctrl graphs')
print ('-dtiff', strcat(DIR, 'res/NCTRL_graphs'))
hold off
input('N-ctrl graphs')

figure()
hold on
for (ii=1:ssize)
    plot(mean(ctrl_matr{ii,2}(:,:)'))

end
title('AvrPTO graphs')
print ('-dtiff', strcat(DIR, 'res/AvrPTO_graphs'))
hold off
input('AvrPTO graphs')

figure()
hold on
for (ii=1:ssize)
    plot(mean(ctrl_matr{ii,3}(:,:)'))

end
title('I96A graphs')
print ('-dtiff', strcat(DIR, 'res/I96A_graphs'))
hold off
input('I96A graphs')

figure()
hold on
for (ii=1:ssize)
    plot(mean(ctrl_matr{ii,4}(:,:)'))

end
title('LUC graphs')
print ('-dtiff', strcat(DIR, 'res/LUC_graphs'))
hold off
input('LUC graphs')
```

```
figure()
hold on
for (ii=1:12)
    for(kk=1:colSize)
    if(tokn_ind_matr(ii,kk)<400 && tokn_ind_matr(ii,kk) )
    plot(mean(slca_matr{ii,tokn_ind_matr(ii,kk)}(:,:)'))
    end
    end
end
title('AvrPTO-probe graphs')
ylabel('Probe')
xlabel('Time')
print ('-dtiff', strcat(DIR, 'res/AvrPTO_probe_graphs'))
hold off
input('AvrPTO-probe graphs')

figure()
hold on
for (ii=13:28)
    for(kk=1:colSize)
    if(tokn_ind_matr(ii,kk)<400 && tokn_ind_matr(ii,kk) )
    plot(mean(slca_matr{ii,tokn_ind_matr(ii,kk)}(:,:)'))
    end
    end
end
title('HopA1-probe graphs')
ylabel('Probe')
xlabel('Time')
print ('-dtiff', strcat(DIR, 'res/HopA1_probe_graphs'))
hold off
input('HopA1-probe graphs')

figure()
hold on
for (ii=29:44)
    for(kk=1:colSize)
    if(tokn_ind_matr(ii,kk)<400 && tokn_ind_matr(ii,kk) )
    plot(mean(slca_matr{ii,tokn_ind_matr(ii,kk)}(:,:)'))
    end
    end
end
title('HopAI1-probe graphs')
ylabel('Probe')
xlabel('Time')
print ('-dtiff', strcat(DIR, 'res/HopAI1_probe_graphs'))
hold off
input('HopAI1-probe graphs')

figure()
hold on
for (ii=45:61)
    for(kk=1:colSize)
    if(tokn_ind_matr(ii,kk)<400 && tokn_ind_matr(ii,kk) )
    plot(mean(slca_matr{ii,tokn_ind_matr(ii,kk)}(:,:)'))
    end
    end
```

```
end
title('HopAF1-probe graphs')
ylabel('Probe')
xlabel('Time')
print ('-dtiff', strcat(DIR, 'res/HopAF1_probe_graphs'))
hold off
input('HopAF1-probe graphs')

figure()
hold on
for (ii=62:63)
    for(kk=1:colSize)
    if(tokn_ind_matr(ii,kk)<400 && tokn_ind_matr(ii,kk) )
    plot(mean(slca_matr{ii,tokn_ind_matr(ii,kk)}(:,:)'))
    end
    end
end
title('HopM1-probe graphs')
ylabel('Probe')
xlabel('Time')
print ('-dtiff', strcat(DIR, 'res/HopM1_probe_graphs'))
hold off
input('HopM1-probe graphs')

figure()
dist_1=pos_ctrl(1,:);
dist_2=pos_ctrl(2,:);
dist_3=pos_ctrl(3,:);
dist_4=pos_ctrl(4,:);
dist_5=pos_matr(:);

boxplot(log10([dist_1', dist_2', dist_3', dist_4', dist_5]))

if (PRINT)
title('Normalized signal distribution')
ylabel('Normalized signal (log10 scale)')
xlabel('[Background, Pto-AvrPto, Pto-AvrPto(I96A), Luciferase, KE Probe]')
print ('-dtiff', strcat(DIR, 'res/SLCA_boxplot'))
end
%%table
str=strcat( DIR , 'res/SLCA_boxplot.xlsx');
tt=tokn_ind_matr;
var1= tt(:) ;
pos=find(var1>0 & var1<400 );
bplot=table([dist_1(pos)', dist_2(pos)', dist_3(pos)', dist_4(pos)', dist_5(pos)]);
writetable(bplot,str);

input('boxplot')


%% PER effector analysis

eff_ind=cell2mat(set2);
p_1=pos_matr(find(eff_ind==1),:);
p_2=pos_matr(find(eff_ind==2),:);
p_3=pos_matr(find(eff_ind==3),:);
```

```
p_4=pos_matr(find(eff_ind==4),:);
p_5=pos_matr(find(eff_ind==5),:);

c_41=pos_ctrl(4,find(eff_ind==1),:);
c_31=pos_ctrl(3,find(eff_ind==1),:);
c_21=pos_ctrl(2,find(eff_ind==1),:);
c_11=pos_ctrl(1,find(eff_ind==1),:);

c_42=pos_ctrl(4,find(eff_ind==2),:);
c_32=pos_ctrl(3,find(eff_ind==2),:);
c_22=pos_ctrl(2,find(eff_ind==2),:);
c_12=pos_ctrl(1,find(eff_ind==2),:);

c_43=pos_ctrl(4,find(eff_ind==3),:);
c_33=pos_ctrl(3,find(eff_ind==3),:);
c_23=pos_ctrl(2,find(eff_ind==3),:);
c_13=pos_ctrl(1,find(eff_ind==3),:);

c_44=pos_ctrl(4,find(eff_ind==4),:);
c_34=pos_ctrl(3,find(eff_ind==4),:);
c_24=pos_ctrl(2,find(eff_ind==4),:);
c_14=pos_ctrl(1,find(eff_ind==4),:);

c_45=pos_ctrl(4,find(eff_ind==5),:);
c_35=pos_ctrl(3,find(eff_ind==5),:);
c_25=pos_ctrl(2,find(eff_ind==5),:);
c_15=pos_ctrl(1,find(eff_ind==5),:);


%%%%%%%%%%%%%%%%%%%%%%%%%
%%  DISPLAY
%%%%%%%%%%%%%%%%%%%%%%%%%

figure()
plot( c_11(:)./c_21(:), p_1(:)./c_21(:), '*g' , c_11(:)./c_21(:), c_41(:)./c_21(:), '*m',↙
c_11(:)./c_21(:), c_21(:)./c_21(:), '*r', c_11(:)./c_21(:), c_31(:)./c_21(:), '*b' )
title('AvrPTO: Normalized Probe vs AvrPTO-CTRL ')
ylabel('Probe')
xlabel('AvrPTO-CTRL')
print ('-dtiff', strcat(DIR, 'res/AvrPto_Probe_AvrPto'))

input('AvrPTO')
figure()
plot( c_12(:)./c_22(:), p_2(:)./c_22(:), '*g' , c_12(:)./c_22(:), c_42(:)./c_22(:), '*m',↙
c_12(:)./c_22(:), c_22(:)./c_22(:), '*r', c_12(:)./c_22(:), c_32(:)./c_22(:), '*b' )
title('HopA1: Normalized Probe vs AvrPTO-CTRL ')
ylabel('Probe')
xlabel('AvrPTO-CTRL')
print ('-dtiff', strcat(DIR, 'res/HopA1_Probe_AvrPto'))

input('HopA1')

figure()
plot( c_13(:)./c_23(:), p_3(:)./c_23(:), '*g' , c_13(:)./c_23(:), c_43(:)./c_23(:), '*m',↙
c_13(:)./c_23(:), c_23(:)./c_23(:), '*r', c_13(:)./c_23(:), c_33(:)./c_23(:), '*b' )
title('HopAI1: Normalized Probe vs AvrPTO-CTRL ')
```

```
ylabel('Probe')
xlabel('AvrPTO-CTRL')
print ('-dtiff', strcat(DIR, 'res/HopAI1_Probe_AvrPto'))

input('HopAI1')

figure()
plot( c_14(:)./c_24(:), p_4(:)./c_24(:), '*g' , c_14(:)./c_24(:), c_44(:)./c_24(:), '*m',↙
c_14(:)./c_24(:), c_24(:)./c_24(:), '*r', c_14(:)./c_24(:), c_34(:)./c_24(:), '*b' )
title('HopAF1: Normalized Probe vs AvrPTO-CTRL ')
ylabel('Probe')
xlabel('AvrPTO-CTRL')
print ('-dtiff', strcat(DIR, 'res/HopAF1_Probe_AvrPto'))

input('HopAF1')

figure()
plot( c_15(:)./c_25(:), p_5(:)./c_25(:), '*g' , c_15(:)./c_25(:), c_45(:)./c_25(:), '*m',↙
c_15(:)./c_25(:), c_25(:)./c_25(:), '*r', c_15(:)./c_25(:), c_35(:)./c_25(:), '*b' )
title('HopM1: Normalized Probe vs AvrPTO-CTRL ')
ylabel('Probe')
xlabel('AvrPTO-CTRL')
print ('-dtiff', strcat(DIR, 'res/HopM1_Probe_AvrPto'))

input('HopM1')


figure()
plot(log10(dist_1),log10(dist_4), '*')
corrcoef(dist_1, dist_4)

if (PRINT)
title('Luciferase vs Background normalized signal')
ylabel('Luciferase (log10 scale)')
xlabel('Background (log10 scale)')
print ('-dtiff', strcat(DIR, 'res/SLCA_CTRL_norm'))
end
%% table
tt=tokn_ind_matr;
ee=eff_ind_matr;
var1= tt(:) ;
pos=find(var1>0 & var1<400 );
var2= ee(:);
pp=log10(dist_1);
hh=log10(dist_4);
val1= pp(:);
val2= hh(:);
str=strcat( DIR , 'res/SLCA_CTRL_norm.txt');
printData22(str, var1(pos), var2(pos), val1(pos) , val2(pos)  );

input('LUC vs N-CTRL norm')

figure()
plot(log10(dist_3),log10(dist_2), '*')
corrcoef(dist_3, dist_2)
```

```
if (PRINT)
title('Pto-AvrPto vs Pto-AvrPto(I96A) normalized signal')
ylabel('Pto-AvrPto (log10 scale)')
xlabel('Pto-AvrPto(I96A) (log10 scale)')
print ('-dtiff', strcat(DIR, 'res/SLCA_FCTRL_norm'))
end

input('AvrPTO vs I96A dist')

figure()
plot(log10(dist_2),log10(dist_4), '*')
corrcoef(dist_2, dist_4)

if (PRINT)
title('Luciferase vs Pto-AvrPto normalized signal')
ylabel('Luciferase (log10 scale)')
xlabel('Pto-AvrPto (log10 scale)')
print ('-dtiff', strcat(DIR, 'res/SLCA_Luc_Pto_norm'))
end
%% table
tt=tokn_ind_matr;
ee=eff_ind_matr;
var1= tt(:) ;
pos=find(var1>0 & var1<400 );
var2= ee(:);
pp=log10(dist_2);
hh=log10(dist_4);
val1= pp(:);
val2= hh(:);
str=strcat( DIR , 'res/SLCA_Luc_Pto_norm.txt');
printData22(str, var1(pos), var2(pos), val1(pos) , val2(pos)  );

input('Luc vs Pto dist')

figure()
plot(log10(dist_3),log10(dist_4), '*')
corrcoef(dist_3, dist_4)

if (PRINT)
title('Luciferase vs Pto-AvrPto(I96A) normalized signal')
ylabel('Luciferase (log10 scale)')
xlabel('Pto-AvrPto(I96A) (log10 scale)')
print ('-dtiff', strcat(DIR, 'res/SLCA_Luc_I96A_norm'))
end
%% table
tt=tokn_ind_matr;
ee=eff_ind_matr;
var1= tt(:) ;
pos=find(var1>0 & var1<400 );
var2= ee(:);
pp=log10(dist_3);
hh=log10(dist_4);
val1= pp(:);
val2= hh(:);
str=strcat( DIR , 'res/SLCA_Luc_I96A_norm.txt');
printData22(str, var1(pos), var2(pos), val1(pos) , val2(pos)  );
```

```
input('Luc vs I96A dist')


figure()
plot(log10(dist_2),log10(dist_5), '*')

if (PRINT)
title('KE Probe vs Pto-AvrPto normalized signal')
ylabel('KE Probe (log10 scale)')
xlabel('Pto-AvrPto (log10 scale)')
print ('-dtiff', strcat(DIR, 'res/SLCA_P_Avr_norm'))
end
%% table
tt=tokn_ind_matr;
ee=eff_ind_matr;
var1= tt(:) ;
pos=find(var1>0 & var1<400 );
var2= ee(:);
pp=log10(dist_2);
hh=log10(dist_5);
val1= pp(:);
val2= hh(:);
str=strcat( DIR , 'res/SLCA_P_Avr_norm.txt');
printData22(str, var1(pos), var2(pos), val1(pos) , val2(pos)  );

input('Probe vs AvrPTO dist')

figure()
plot(log10(dist_3),log10(dist_5), '*')

if (PRINT)
title('KE Probe vs Pto-AvrPto(I96A) normalized signal')
ylabel('KE Probe (log10 scale)')
xlabel('Pto-AvrPto(I96A) (log10 scale)')
print ('-dtiff', strcat(DIR, 'res/SLCA_P_I96A_norm'))
end
%% table
tt=tokn_ind_matr;
ee=eff_ind_matr;
var1= tt(:) ;
pos=find(var1>0 & var1<400 );
var2= ee(:);
pp=log10(dist_3);
hh=log10(dist_5);
val1= pp(:);
val2= hh(:);
str=strcat( DIR , 'res/SLCA_P_I96A_norm.txt');
printData22(str, var1(pos), var2(pos), val1(pos) , val2(pos)  );

input('Probe vs I96A dist')


%% Scatter diagrams on bio repeats
%% Probe scatter
```

```
figure()
x=d_matr( 1 ,:, :, 1 )./mean(c_matr( 2 , :, :, : ),4);
y=d_matr( 1 , :, :, 2 )./mean(c_matr( 2 , :, :, : ),4);
corrcoef(x,y)

plot(x(:),y(:), '*')

if (PRINT)
title('Probe T2 vs T1')
ylabel('T2')
xlabel('T1')
print ('-dtiff', strcat(DIR, 'res/SLCA_P_scatternm1'))
end
%% table
tt=tokn_ind_matr;
ee=eff_ind_matr;
var1= tt(:) ;
pos=find(var1>0 & var1<400 );
var2= ee(:);
pp=x(:);
hh=y(:);
val1= pp(:);
val2= hh(:);
str=strcat( DIR , 'res/SLCA_P_scatternm1.txt');
printData22(str, var1(pos), var2(pos), val1(pos) , val2(pos)  );

input('Probe scatter nm1')

%%%%%

figure()
x=d_matr( 1 ,:, :, 2 )./mean(c_matr( 2 , :, :, : ),4);
y=d_matr( 1 , :, :, 3 )./mean(c_matr( 2 , :, :, : ),4);
corrcoef(x,y)

plot(x(:),y(:), '*')

if (PRINT)
title('Probe T3 vs T2')
ylabel('T3')
xlabel('T2')
print ('-dtiff', strcat(DIR, 'res/SLCA_P_scatternm2'))
end

input('Probe scatter nm2')


figure()
x=d_matr( 1 ,:, :, 3 )./mean(c_matr( 2 , :, :, : ),4);
y=d_matr( 1 , :, :, 4 )./mean(c_matr( 2 , :, :, : ),4);
corrcoef(x,y)
plot(x(:),y(:), '*')

if (PRINT)
title('Probe T4 vs T3')
ylabel('T4')
```

```
xlabel('T3')
print ('-dtiff', strcat(DIR, 'res/SLCA_P_scatternm3'))
end
%% table
tt=tokn_ind_matr;
ee=eff_ind_matr;
var1= tt(:) ;
pos=find(var1>0 & var1<400 );
var2= ee(:);
pp=x(:);
hh=y(:);
val1= pp(:);
val2= hh(:);
str=strcat( DIR , 'res/SLCA_P_scatternm3.txt');
printData22(str, var1(pos), var2(pos), val1(pos) , val2(pos)  );
input('Probe scatter nm3')

figure()
x=d_matr( 1 ,:, :, 4 )./mean(c_matr( 2 , :, :, : ),4);
y=d_matr( 1 , :, :, 1 )./mean(c_matr( 2 , :, :, : ),4);
corrcoef(x,y)

plot(x(:),y(:), '*')

if (PRINT)
title('Probe T1 vs T4')
ylabel('T1')
xlabel('T4')
print ('-dtiff', strcat(DIR, 'res/SLCA_P_scatternm4'))
end

input('Probe scatter nm4')


%% CTRL scatter
%%%%%%%%%%
figure()
x=c_matrn( 1, :, :, 1 );
y=c_matrn( 1, :, :, 2 );
plot(x(:),y(:), '*')

if (PRINT)
title('I96A T2 vs T1')
ylabel('T2')
xlabel('T1')
print ('-dtiff', strcat(DIR, 'res/SLCA_I96A_scatter_n1'))
end

input('I96A scatter n1')

figure()
x=c_matrn( 1, :, :, 2 );
y=c_matrn( 1, :, :, 3 );
plot(x(:),y(:), '*')

if (PRINT)
```

```
title('I96A T3 vs T2')
ylabel('T3')
xlabel('T2')
print ('-dtiff', strcat(DIR, 'res/SLCA_I96A_scatter_n2'))
end

input('I96A scatter n2')

figure()
x=c_matrn( 1, :, :, 3 );
y=c_matrn( 1, :, :, 4 );
plot(x(:),y(:), '*')

if (PRINT)
title('I96A T4 vs T3')
ylabel('T4')
xlabel('T3')
print ('-dtiff', strcat(DIR, 'res/SLCA_I96A_scatter_n3'))
end

input('I96A scatter n3')


figure()
x=c_matrn( 1, :, :, 4 );
y=c_matrn( 1, :, :, 1 );
plot(x(:),y(:), '*')

if (PRINT)
title('I96A T1 vs T4')
ylabel('T1')
xlabel('T4')
print ('-dtiff', strcat(DIR, 'res/SLCA_I96A_scatter_n4'))
end

input('I96A scatter n4')

figure()
scatter3(v3(:), v2(:), v1(:), 'filled')
if (PRINT)
title('3D scatterplot of controls')
zlabel('I96A')
ylabel('AvrPto')
xlabel('Luc')
print ('-dtiff', strcat(DIR, 'res/Scatter3D_Luc_AvrPto_I96A'))
end
input('3D scatter of controls')

%% AvrPTO
%%%%%%%%%%

figure()
x=c_matrn( 2, :, :, 1 );
y=c_matrn( 2, :, :, 2 );
plot(x(:),y(:), '*')
```

```
if (PRINT)
title('AvrPTO T2 vs T1')
ylabel('T2')
xlabel('T1')
print ('-dtiff', strcat(DIR, 'res/SLCA_AvrPTO_scatter1'))
end
input('AvrPTO scatter 1')

figure()
x=c_matrn( 2, :, :, 3 );
y=c_matrn( 2, :, :, 4 );
plot(x(:),y(:), '*')

if (PRINT)
title('AvrPTO T4 vs T3')
ylabel('T4')
xlabel('T3')
print ('-dtiff', strcat(DIR, 'res/SLCA_AvrPTO_scatter3'))
end
input('AvrPTO scatter 2')

%%End scatter

%%%%%%%%%%%%%%%%%%%%%%%%%%%%%%%%%%%%%%%%%%%%%%%
%% Testing normalized data cross experiments
%%%%%%%%%%%%%%%%%%%%%%%%%%%%%%%%%%%%%%%%%%%%%%%

for (ii=1:ssize)

%% testing
%%%%
%% Probe-adjusted I96A
cc3=c_matr( 3 , ii, kk, : );
cc2=c_matr( 2 , ii, kk, : );
I96A_adj=predict(lm, [cc3(:) cc2(:)]);

    for(kk=1:colSize)
        dd=d_matr( 1 , ii, kk, : );
        [HVal(ii,kk),pVal(ii,kk)]=ttest2(dd(:) , I96A_adj(:)  , 0.05, 'right');

        %% Average values for the Controls and probes
        m_Val (ii,kk) = mean(dd(:)');
        std_Val(ii,kk) = std(dd(:)');
        I96_std_Val(ii,kk)= std(I96A_adj(:)');
        I96_m_Val(ii,kk)= mean(I96A_adj(:)');

    end


%%% Pool the I96, AvrPto and estimate ctrl
    c_31=c_matr(3,find(eff_ind==cell2mat(set2(ii))),1,:);
    c_21=c_matr(2,find(eff_ind==cell2mat(set2(ii))),1,:);
    I96A_adj_c=predict(lm, [c_31(:) c_21(:)]);

%% estimate
```

```
    for(kk=1:colSize)
        dd=d_matr( 1 , ii, kk, : )  ;
        [HVal_n(ii,kk),pVal_n(ii,kk)] = ttest2(dd(:), I96A_adj_c  ,  0.05, 'right');


    end
end

%%%%
%% Nomalized and pooled CTRL and effectors
Avr_std_Val=zeros(5,1);
Avr_m_Val=zeros(5,1);
Luc_std_Val=zeros(5,1);
I96A_std_Val=zeros(5,1);
Luc_m_Val=zeros(5,1);
I96A_m_Val=zeros(5,1);


for (kk=1:5)
    c_11=c_matrn(1,find(eff_ind==kk),1,:);
    c_21=c_matrn(2,find(eff_ind==kk),1,:);
    c_31=c_matrn(3,find(eff_ind==kk),1,:);

        I96A_std_Val(kk)= std(c_11(:)');
        I96A_m_Val(kk)= mean(c_11(:)');
        Avr_std_Val(kk)= std(c_21(:)');
        Avr_m_Val(kk)= mean(c_21(:)');
        Luc_std_Val(kk)= std(c_31(:)');
        Luc_m_Val(kk)= mean(c_31(:)');
end


for (ii=1:ssize)

%%% Pool the I96 ctrl
    c_11=c_matrn(1,find(eff_ind==cell2mat(set2(ii))),1,:);
    c_21=c_matrn(2,find(eff_ind==cell2mat(set2(ii))),1,:);
%% Estimate

    min_p=min(find(eff_ind==cell2mat(set2(ii))));
    tki=tokn_ind_matr(find(eff_ind==cell2mat(set2(ii))),:);

    for(kk=1:colSize)
        if ( tokn_ind_matr(ii,kk) >0 && tokn_ind_matr(ii,kk) < 400 )
        [pos_k1, pos_k2]= find(tki==tokn_ind_matr(ii, kk));
        dd=[]; dd3=[];

        for(mm=1:size(pos_k1,1))
        dd=[ dd, (d_matr( 1 , pos_k1(mm)+ min_p-1, pos_k2(mm), : )./mean(c_matr( 3 ,↙
pos_k1(mm)+ min_p-1, pos_k2(mm), : ),4))-c_matrn( 1 , pos_k1(mm)+ min_p-1, pos_k2(mm), :↙
) ] ;
        dd3=[ dd3, (d_matr( 3 , pos_k1(mm)+ min_p-1, pos_k2(mm), : )./mean(c_matr( 3 ,↙
pos_k1(mm)+ min_p-1, pos_k2(mm), : ),4))-c_matrn( 1 , pos_k1(mm)+ min_p-1, pos_k2(mm), :↙
) ] ;
        end
```

```
        %% Average values for the Controls and probes
        I_Val(ii,kk) = mean(dd3(:)');

        [HVal_np(ii,kk),pVal_np(ii,kk)] = ttest2( dd(:) , (c_21(:)-c_11(:))*k_ctrl ,↙
0.05, 'right');
        end
    end
end


%%%%%%%%%%%%%%%%%%
%% FDR correction
%%%%%%%%%%%%%%%%%%
%%MAFDR
[pVal_fdr, qVal(:), pi0]=mafdr (pVal(:) );
input('next')
%%%%%% End FDR

%%%%%%%%%%%%%%%%%%%%%%%%%%%%%%%%%%%%%%%%%%%%
%% Output the pValues
tt=tokn_ind_matr';
ee=eff_ind_matr';
var1= tt(:) ;
pos=find(var1>0 & var1<400 );
var2= ee(:);
pp=pVal';
hh=HVal';
val1= pp(:);
val2= hh(:);
str=strcat( DIR , 'res/SLCA_pVal_hits.txt');
printData22(str, var1(pos), var2(pos), val1(pos) , val2(pos)  );

%%%%%%%%%%%%%%%%%%%%%%%%%%%%%%%%%%%%%%%%%%%%
%% Output the pValues with norm
tt=tokn_ind_matr';
ee=eff_ind_matr';
var1= tt(:) ;
pos=find(var1>0 & var1<400 );
var2= ee(:);
pp=pVal_n';
hh=HVal_n';
val1= pp(:);
val2= hh(:);
str=strcat( DIR , 'res/SLCA_pVal_norm_hits.txt');
printData22(str, var1(pos), var2(pos), val1(pos) , val2(pos)   );

%%%%%%%%%%%%%%%%%%%%%%%%%%%%%%%%%%%%%%%%%%%%
%% Output the  qValues
tt=tokn_ind_matr';
ee=eff_ind_matr';
pp=plateID';
var1= tt(:) ;
pos=find(var1>0 & var1<400 );
var2= ee(:);
var3=pp(:);
pp=( m_Val./I96_m_Val )';
```

```
hh=qVal';
hh1=pVal';
hh2=pVal_n';
%%hh=HVal_np';
val1= pp(:);
val2= hh(:);
val3= hh1(:);
val4= hh2(:);
str=strcat( DIR , 'res/SLCA_qVal_all.txt');
printData24(str, var1(pos), var2(pos), var3(pos), val1(pos) , val2(pos), val3(pos), val4↙
(pos)   );


%%%%%%%%%%%%%%%%%%%%%%%%%%%%%%%%%%%%%%%%%%%%
%% Output the hits
tt=tokn_ind_matr';
ee=eff_ind_matr';
pp=plateID';
var1= tt(:) ;
pos=find(var1>0 & var1<400 );
var2= ee(:);
var3=pp(:);
pp=( m_Val./I96_m_Val )';
hh=qVal';
hh1=pVal';
hh2=pVal_n';
val1= pp(:);
val2= hh(:);
val3= hh1(:);
val4= hh2(:);
hitMatr=[num2cell(var1(pos)) cellstr(var2(pos)) cellstr(var3(pos)) num2cell(val1(pos))↙
num2cell(val2(pos)) num2cell(val3(pos)) num2cell(val4(pos)) ] ;
[hMatr, I_hMatr]=sortrows(hitMatr, [2  1 5 ] );
hSize=size(hitMatr,1);
hMatr_s=[];
for (ii=1:hSize)
if( (ii==1 || cell2mat(hMatr(ii,1))~=cell2mat(hMatr(ii-1,1))) && cell2mat(hMatr(ii,5))<0.↙
05)
    hMatr_s=[hMatr_s ; hMatr(ii,:)];
end
end
str=strcat( DIR , 'res/SLCA_qVal_hit_list.txt');
printData22(str, cell2mat(hMatr_s(:,1)), hMatr_s(:,2), cell2mat(hMatr_s(:,4)) ,  cell2mat↙
(hMatr_s(:,5))   );



%%%%%%%%%%%%%%%%%%%%%%%%%%%%%%%%%%%%%%%%%%%%%%%%%%%%%%%%%%%%%%%%%%%%%%%%%%%%%%%%%%%%%%%%%%↙
%%%%%%%%%%%%%%%%%%%%%%%
input('done')
%%%%%%%%%%%%%%%%%%%%%%%%%%%%%%%%%%%%%%%%%%%%%%%%%%%%%%%%%%%%%%%%%%%%%%%%%%%%%%%%%%%%%%%%%%↙
%%%%%%%%%%%%%%%%%%%%%%%%
```

```
function printData22( str,  var1, var2, score1 , score2  )

fid = fopen(str,'w');
for i=1:size(var1,1)
     fprintf(fid,'%i \t  %s  \t %e \t %e    ', var1(i,:),  cell2mat(var2(i,:)) , score1↙
(i), score2(i)  ) ;
     fprintf(fid,'\n') ;
end
fclose(fid)
```

```
function printData24( str,  var1, var2, var3, score1 , score2, score3, score4  )

fid = fopen(str,'w');
for i=1:size(var1,1)
     fprintf(fid,'%i \t  %s  \t %s \t %e \t %e \t %e \t %e  ', var1(i,:),  cell2mat(var2↙
(i,:)) , cell2mat(var3(i,:)) , score1(i), score2(i) ,  score3(i) ,  score4(i)  ) ;
     fprintf(fid,'\n') ;
end
fclose(fid)
```

```
function [DIR,str_cell, set1, set2]=load_SLCA(param)

%%EFFECTORS SET
set1={'AvrPto', 'HopA1', 'HopAI1','HopAF1', 'HopM1'};

%% TOKN indexing:  PTO:400; BAK1:401; FLS2:402; FEN:403

switch (param)
%%%%%%%%%%%%%%%%%%%%
%%% SLCA DATA SETS
%%%%%%%%%%%%%%%%%%%
%% Biological replicates

case 1
DIR='../data/SLCA/';


%% Files
%% HopA1
slca1='011014_HopA1.xlsx' ;
slca2='021113_HopA1.xlsx' ;
slca3='022013_HopA1.xlsx' ;
slca4='031613_HopA1.xlsx' ;
slca5='040313_HopA1.xlsx' ;
slca6='042613_HopA1.xlsx' ;
slca7='071013_HopA1.xlsx' ;
slca8='072112_HopA1.xlsx' ;
slca9='072512_HopA1.xlsx' ;
slca10='080712_HopA1.xlsx' ;
slca11='092012_HopA1.xlsx' ;
slca12='092613_HopA1.xlsx' ;
slca13='101212_HopA1.xlsx' ;
slca14='120112_HopA1.xlsx' ;
slca15='120712_HopA1.xlsx' ;

str_cell= {slca1, slca2, slca3, slca4, slca5, slca6, slca7, slca8, slca9, slca10, slca11,↙
slca12, slca13, slca14, slca15};
set2={2, 2, 2, 2, 2, 2, 2, 2, 2, 2, 2, 2, 2, 2, 2 };

case 2
DIR='../data/SLCA/';


%% Effector index

%% FILES
%% AvrPto: 12 plates
slca1='120712_AvrPto.xlsx' ;
slca3='102212_AvrPto.xlsx' ;
slca4='101212_AvrPto.xlsx' ;
slca5='092012_AvrPto.xlsx' ;
slca7='071013_AvrPto.xlsx' ;
slca9='062513_AvrPto.xlsx' ;
slca10='050813_AvrPto.xlsx' ;
slca12='031613_AvrPto.xlsx' ;
slca13='022013_AvrPto.xlsx' ;
```

```
slca14='021113_AvrPto.xlsx' ;
slca87='121913_AvrPto_m.xlsx' ;
slca88='092613_AvrPto_m.xlsx' ;

%% HopA1: 16 plates
slca16='050813_HopA1.xlsx' ;
slca17='120712_HopA1.xlsx' ;
slca18='120112_HopA1.xlsx' ;
slca21='092012_HopA1.xlsx' ;
slca22='072512_HopA1.xlsx' ;
slca23='072112_HopA1.xlsx' ;
slca24='071013_HopA1.xlsx' ;
slca26='031613_HopA1.xlsx' ;
slca27='022013_HopA1.xlsx' ;
slca28='021113_HopA1.xlsx' ;
slca30='042613_HopA1.xlsx' ;
slca49='121913_HopA1.xlsx' ;
slca50='062513_HopA1.xlsx' ;
slca91='080712_HopA1_m.xlsx' ;
slca92='121913_HopA1_m.xlsx' ;
slca101='050814_HopA1.xlsx' ;

%% HopAI1: 16 plates
slca31='050813_HopAI1.xlsx' ;
slca32='051813_HopAI1_a.xlsx' ;
slca33='051813_HopAI1_b.xlsx' ;
slca35='052412_HopAI1_a.xlsx' ;
slca37='052412_HopAI1_b.xlsx' ;
slca39='052412_HopAI1_c.xlsx' ;
slca40='071013_HopAI1.xlsx' ;
slca41='040313_HopAI1.xlsx' ;
slca42='031613_HopAI1.xlsx' ;
slca43='011014_HopAI1.xlsx' ;
slca44='042613_HopAI1_a.xlsx' ;
slca45='042613_HopAI1_b.xlsx' ;
slca46='121913_HopAI1.xlsx' ;
slca47='092613_HopAI1.xlsx' ;
slca48='062513_HopAI1.xlsx' ;
slca94='092613_HopAI1_m.xlsx' ;

%% HopAF1: 17 plates
slca52='120712_HopAF1.xlsx' ;
slca53='082912_HopAF1.xlsx' ;
slca54='050813_HopAF1.xlsx' ;
slca55='051813_HopAF1_a.xlsx' ;
slca56='051813_HopAF1_b.xlsx' ;
slca58='102212_HopAF1.xlsx' ;
slca61='092012_HopAF1.xlsx' ;
slca64='040313_HopAF1.xlsx' ;
slca65='031613_HopAF1.xlsx' ;
slca66='022013_HopAF1.xlsx' ;
slca67='021113_HopAF1.xlsx' ;
slca68='011014_HopAF1.xlsx' ;
slca69='121913_HopAF1.xlsx' ;
slca70='062513_HopAF1.xlsx' ;
slca71='042613_HopAF1.xlsx' ;
```

```
slca83='080712_HopAF1.xlsx' ;
slca96='092613_HopAF1_m.xlsx' ;

%% HopM1: 2 plates
slca86='082912_HopM1.xlsx' ;
slca97='080712_HopM1_m.xlsx' ;

%% PUBLISHED MPMI
slca99='111712_Pilot.xlsx' ;
slca100='021413b_Pilot.xlsx' ;

str_cell= {slca1,  slca3, slca4, slca5, slca7,      slca9, slca10, slca12, slca13,↙
slca14, slca87, slca88,           slca16, slca17, slca18,       slca21, slca22, slca23,↙
slca24, slca26, slca27, slca28,        slca30, slca49, slca50, slca91, slca92, slca101,↙
slca31, slca32, slca33, slca35, slca37,        slca39, slca40, slca41, slca42, slca43,↙
slca44, slca45, slca46, slca47, slca48, slca94,        slca52, slca53, slca54, slca55,↙
slca56,      slca58, slca61,    slca64, slca65, slca66, slca67, slca68, slca69, slca70,↙
slca71,  slca83, slca96,     slca86, slca97 };
set2={ 1, 1 , 1, 1, 1, 1, 1, 1, 1, 1, 1, 1,      2, 2, 2, 2, 2, 2, 2, 2, 2, 2, 2, 2, 2,↙
2, 2, 2,   3, 3, 3, 3, 3, 3, 3, 3, 3, 3, 3, 3, 3, 3 ,3, 3,   4, 4, 4, 4, 4, 4, 4, 4, 4,↙
4, 4, 4, 4, 4, 4, 4, 4,  5, 5  };

end
```
