# Supplementary material for: Integrative network-centric approach reveals signaling pathways associated with plant resistance and susceptibility to Pseudomonas syringae
Source: PLoS Biol. 2018 Dec 12;16(12):e2005956. doi: 10.1371/journal.pbio.2005956 (PMC6322785; doi:10.1371/journal.pbio.2005956)
Supplement: S1 Table — K-E, kinase–effector; KEI, Kinase Effector Interactor. (DOCX) [file pbio.2005956.s010.docx]

**Supplementary Table 1**: A list of KEIS including the interacting effector and the calculated interaction strength (delta). The list was used to generate the Kinase-Effector network from Figure 2A.

| **KEI#** | **Effector** | **Fold change vs. controls** |
| --- | --- | --- |

6 HopAI1 1.61E+00

7 AvrPto 2.23E+00

7 HopA1 6.36E+00

7 HopAF1 1.90E+00

7 HopAI1 5.94E+00

9 AvrPto 4.50E+00

9 HopA1 1.16E+01

9 HopAF1 8.39E+00

9 HopAI1 2.10E+01

10 AvrPto 1.33E+00

10 HopA1 5.33E+00

10 HopAF1 2.59E+00

10 HopAI1 9.84E+00

11 AvrPto 6.75E+00

11 HopA1 1.29E+01

11 HopAF1 1.23E+01

11 HopAI1 1.82E+00

20 AvrPto 1.03E+01

20 HopA1 1.75E+01

20 HopAF1 1.56E+01

20 HopAI1 2.70E+01

25 HopA1 9.03E+00

26 HopA1 2.69E+00

31 HopA1 6.36E+00

31 HopAF1 2.43E+00

31 HopAI1 6.19E+00

33 HopA1 2.35E+00

33 HopAI1 2.22E+00

35 HopA1 1.52E+00

37 AvrPto 3.19E+00

37 HopA1 8.94E+00

37 HopAF1 7.03E+00

37 HopAI1 1.60E+01

38 HopA1 2.08E+00

38 HopAI1 3.51E+00

40 HopA1 2.75E+00

40 HopAF1 2.44E+00

40 HopAI1 8.10E+00

44 HopA1 2.73E+00

44 HopAI1 5.55E+00

45 HopA1 3.58E+00

45 HopAF1 1.54E+00

45 HopAI1 5.30E+00

47 HopAI1 1.94E+00

51 HopA1 5.56E+00

51 HopAF1 1.25E+00

51 HopAI1 3.98E+00

52 HopA1 6.94E+00

52 HopAF1 2.62E+00

52 HopAI1 5.07E+00

54 HopA1 1.62E+00

54 HopAI1 3.21E+00

63 AvrPto 3.39E+00

63 HopA1 8.25E+00

63 HopAF1 6.13E+00

63 HopAI1 5.93E+00

67 AvrPto 2.79E+01

67 HopA1 3.42E+01

67 HopAF1 1.64E+01

67 HopAI1 3.48E+01

72 HopA1 4.77E+00

72 HopAF1 5.64E+00

72 HopAI1 5.27E+00

73 AvrPto 9.96E+00

73 HopA1 1.69E+01

73 HopAF1 2.14E+01

73 HopAI1 5.53E+00

80 HopAI1 1.70E+00

86 HopA1 1.69E+00

86 HopAF1 4.21E+00

86 HopAI1 9.46E+00

89 AvrPto 2.72E+00

89 HopA1 1.04E+01

89 HopAF1 3.49E+00

89 HopAI1 6.45E+00

91 HopA1 1.78E+01

91 HopAF1 4.79E+00

91 HopAI1 1.14E+01

92 AvrPto 2.26E+00

92 HopA1 8.74E+00

92 HopAF1 5.30E+00

92 HopAI1 1.36E+01

104 AvrPto 4.03E+00

104 HopA1 1.83E+01

104 HopAF1 5.23E+00

104 HopAI1 8.29E+00

105 HopA1 1.38E+00

105 HopAF1 2.05E+00

113 HopAI1 1.76E+00

119 HopA1 2.83E+00

120 HopA1 4.10E+00

120 HopAF1 1.66E+00

143 AvrPto 1.97E+00

143 HopA1 3.57E+00

143 HopAF1 5.03E+00

143 HopAI1 6.67E+00

147 HopA1 1.61E+00

147 HopAF1 1.61E+00

147 HopAI1 3.63E+00

149 AvrPto 1.20E+00

149 HopA1 4.74E+00

149 HopAF1 1.79E+00

149 HopAI1 5.10E+00

150 HopA1 2.65E+00

150 HopAF1 2.81E+00

151 HopAF1 3.85E+00

151 HopAI1 3.11E+00

152 HopA1 1.58E+00

152 HopAI1 4.05E+00

153 AvrPto 1.47E+00

153 HopA1 3.80E+00

153 HopAF1 2.67E+00

153 HopAI1 6.31E+00

154 HopAI1 2.67E+00

156 AvrPto 1.40E+00

156 HopA1 6.58E+00

156 HopAF1 1.75E+00

156 HopAI1 4.88E+00

158 HopA1 2.11E+00

158 HopAF1 1.35E+00

158 HopAI1 1.50E+00

160 AvrPto 3.87E+00

160 HopA1 6.08E+00

160 HopAF1 3.43E+00

160 HopAI1 5.77E+00

161 AvrPto 2.62E+00

161 HopA1 1.08E+01

161 HopAI1 3.58E+00

163 AvrPto 3.56E+00

163 HopA1 1.72E+01

163 HopAF1 9.84E+00

163 HopAI1 1.17E+01

164 HopA1 3.76E+00

164 HopAI1 3.65E+00

188 AvrPto 1.78E+00

188 HopA1 5.83E+00

188 HopAF1 4.50E+00

188 HopAI1 1.17E+01

192 HopAI1 3.14E+00

194 HopA1 3.74E+00

194 HopAF1 2.04E+00

194 HopAI1 5.84E+00

195 AvrPto 5.91E+00

196 AvrPto 3.53E+00

196 HopA1 1.75E+01

196 HopAF1 1.37E+01

196 HopAI1 3.04E+01

201 HopA1 2.16E+00

201 HopAF1 4.15E+00

201 HopAI1 7.67E+00

206 HopAI1 1.45E+00

209 HopA1 1.76E+00

209 HopAI1 1.81E+00

220 AvrPto 2.06E+00

220 HopA1 1.06E+01

220 HopAF1 3.15E+00

220 HopAI1 7.36E+00

221 AvrPto 2.90E+00

221 HopA1 7.02E+00

221 HopAF1 4.50E+00

221 HopAI1 1.32E+01

223 HopA1 3.27E+00

223 HopAF1 2.27E+00

223 HopAI1 2.59E+00

229 HopA1 1.67E+00

229 HopAI1 1.48E+00

235 HopA1 5.14E+00

235 HopAI1 3.30E+00

237 AvrPto 2.46E+00

237 HopA1 4.52E+00

237 HopAF1 6.82E+00

237 HopAI1 6.30E+00

244 HopAI1 2.00E+00

245 AvrPto 2.61E+00

245 HopA1 7.23E+00

245 HopAF1 2.84E+00

245 HopAI1 1.88E+01

247 HopA1 1.39E+00

248 HopA1 3.54E+00

248 HopAI1 1.49E+00

250 HopA1 4.49E+00

250 HopAI1 1.90E+00

251 AvrPto 1.42E+00

251 HopA1 3.64E+00

251 HopAF1 2.36E+00

251 HopAI1 4.54E+00

252 HopAI1 2.00E+00

255 HopA1 2.51E+00

255 HopAF1 1.43E+00

257 HopA1 2.44E+00

259 HopA1 3.87E+00

259 HopAF1 1.56E+00

259 HopAI1 2.65E+00

260 HopA1 2.15E+00

263 HopAI1 2.93E+00

264 HopA1 3.82E+00

266 HopAI1 5.45E+00

267 HopA1 2.43E+00

271 HopA1 3.83E+00

271 HopAI1 4.47E+00

272 HopA1 5.05E+00

272 HopAF1 1.65E+00

272 HopAI1 1.51E+00

273 HopA1 2.23E+00

273 HopAI1 3.41E+00

274 AvrPto 1.88E+00

274 HopA1 9.36E+00

274 HopAF1 4.13E+00

274 HopAI1 1.20E+01

275 HopA1 2.30E+00

275 HopAI1 1.65E+00

278 HopA1 1.19E+00

279 HopA1 1.27E+01

279 HopAF1 3.29E+00

279 HopAI1 2.93E+00

285 HopA1 1.85E+00

285 HopAI1 1.61E+00

286 HopA1 2.44E+00

289 HopA1 1.32E+00

289 HopAF1 2.47E+00

289 HopAI1 1.93E+00

290 HopA1 1.99E+00

290 HopAI1 3.44E+00

293 HopA1 1.52E+00

295 HopA1 2.48E+00

298 HopAI1 1.87E+00

299 HopA1 5.48E+00

299 HopAF1 2.83E+00

299 HopAI1 2.10E+00

301 AvrPto 9.72E+00

301 HopA1 2.19E+01

301 HopAF1 7.80E+01

301 HopAI1 2.13E+02

302 HopA1 1.26E+00

304 AvrPto 3.88E+00

304 HopA1 1.28E+01

304 HopAF1 7.16E+00

304 HopAI1 9.04E+00

305 AvrPto 1.56E+00

305 HopA1 3.23E+00

311 HopA1 1.48E+00

311 HopAI1 1.72E+00

313 AvrPto 2.39E+00

313 HopA1 1.00E+01

313 HopAF1 5.33E+00

313 HopAI1 5.33E+00

315 HopA1 2.78E+00

315 HopAI1 4.32E+00

318 AvrPto 2.45E+00

318 HopA1 1.19E+01

318 HopAF1 7.18E+00

318 HopAI1 1.24E+01

319 HopAI1 3.34E+00

322 HopA1 2.62E+00

323 AvrPto 1.49E+00

323 HopA1 1.03E+01

323 HopAF1 5.43E+00

323 HopAI1 2.67E+00

324 HopA1 2.40E+00

324 HopAF1 1.40E+00

324 HopAI1 8.76E+00

326 AvrPto 1.46E+00

326 HopA1 5.51E+00

326 HopAF1 2.87E+00

326 HopAI1 9.18E+00

327 AvrPto 3.58E+00

327 HopA1 9.36E+00

327 HopAF1 6.21E+00

327 HopAI1 3.47E+01

329 HopA1 4.63E+00

338 HopA1 3.03E+00

338 HopAI1 1.75E+00

339 HopA1 9.44E+00

339 HopAF1 4.48E+00

339 HopAI1 7.24E+00

339 AvrPto 1.93E+00

342 HopAF1 4.48E+00

342 HopAI1 1.06E+01

345 HopA1 2.14E+00

348 AvrPto 3.93E+00

348 HopA1 1.94E+00

348 HopAF1 1.93E+00

348 HopAI1 3.53E+00

350 HopA1 1.82E+00

355 HopA1 3.66E+00

355 HopAF1 1.70E+00

355 HopAI1 1.92E+00

357 HopA1 1.81E+00

357 HopAI1 4.66E+00

361 AvrPto 1.85E+00

361 HopA1 4.20E+00

361 HopAF1 2.46E+00

361 HopAI1 9.03E+00

365 AvrPto 3.38E+00

366 HopA1 9.98E+00

366 HopAF1 2.26E+00

366 HopAI1 1.83E+00

368 HopAI1 2.78E+00

376 HopA1 3.11E+01

376 HopAF1 1.65E+01

376 HopAI1 4.31E+01

377 AvrPto 2.36E+00

377 HopA1 9.18E+00

377 HopAF1 5.50E+00

377 HopAI1 6.21E+00

379 HopA1 4.71E+00

379 HopAF1 1.91E+00

379 HopAI1 2.31E+00

381 HopA1 1.30E+00

382 HopA1 4.26E+00

383 HopA1 2.17E+00

383 HopAF1 3.94E+00

383 HopAI1 1.17E+01

384 HopAF1 1.06E+01

384 HopAI1 2.81E+01

385 HopA1 3.95E+00

387 HopAI1 1.46E+00

389 AvrPto 9.76E+00

389 HopA1 3.79E+01

389 HopAF1 5.20E+00

389 HopAI1 6.17E+00
