# Supplementary material for: Integrative network-centric approach reveals signaling pathways associated with plant resistance and susceptibility to Pseudomonas syringae
Source: PLoS Biol. 2018 Dec 12;16(12):e2005956. doi: 10.1371/journal.pbio.2005956 (PMC6322785; doi:10.1371/journal.pbio.2005956)
Supplement: S2 Table — BLAST, Basic Local Alignment Search Tool; KEI, Kinase Effector Interactor. (DOCX) [file pbio.2005956.s011.docx]

**Supplemental Table 2:** A list of the focus KEIs including the KEI number, <https://solgenomics.net/> ID, the top BLAST hit in the Arabidopsis genome, known symbol, kinase structural class in PlantsP database (<http://plantsp.sdsc.edu>) and their bacterial effector interactors.

| **KEI#** | **ID (SolLyc)** | **NCBI ID** | **Top BLAST hit Arabidopsis** | **Symbol** | **Kinase Class** | **Interactors** | | | |
| --- | --- | --- | --- | --- | --- | --- | --- | --- | --- |
| 7 | Solyc07g042590 | LOC101266653 | AT2G07180 | PBL8 | PPC:1.2.2 | AvrPto | HopA1 | HopAF1 | HopAI1 |
| 20 | Solyc01g097980 | TCTR2 (LeCTR2) | AT1G08720 | EDR1 | PPC:2.1.3 | AvrPto | HopA1 | HopAF1 | HopAI1 |
| 25 | Solyc02g078140 | LOC101252042 | AT5G58950 | RAF36 | PPC:2.1.4 |  | HopA1 |  |  |
| 33 | Solyc06g082440 | CIPK11 | AT2G30360 | CIPK11 | PPC:4.2.4 |  | HopA1 |  | HopAI1 |
| 37 | Solyc02g065520 | LOC100736543 | AT2G33580 | LYK10 | PPC:1.1.3 | AvrPto | HopA1 | HopAF1 | HopAI1 |
| 72 | Solyc06g071810 | LOC101259548 | AT2G31880 |  | PPC:1.12.5 |  | HopA1 | HopAF1 | HopAI1 |
| 86 | Solyc11g072660 | LOC101254270 | AT1G07870 | PBL5 | PPC:1.2.2 |  | HopA1 | HopAF1 | HopAI1 |
| 91 | Solyc03g113450 | LOC101253104 | AT5G62710 |  | PPC:1.12.2 |  | HopA1 | HopAF1 | HopAI1 |
| 92 | Solyc05g056370 | LOC101244667 | AT5G63710 |  | PPC:1.12.2 | AvrPto | HopA1 | HopAF1 | HopAI1 |
| 104 | Solyc11g064890 | LOC543567 | AT1G63500 | BSK7 or BSK8 | PPC:1.16.1 | AvrPto | HopA1 | HopAF1 | HopAI1 |
| 143 | Solyc02g081070 | LOC101245221 | AT4G22130 | SRF8 | PPC:1.1.1 | AvrPto | HopA1 | HopAF1 | HopAI1 |
| 149 | Solyc03g006890 | LOC101267580 | AT3G15890 | PTI1-like | PPC:1.10.1 |  | HopA1 | HopAF1 | HopAI1 |
| 150 | Solyc02g068300 | LecRK | AT5G06740 |  | PPC:1.11.1 |  | HopA1 | HopAF1 |  |
| 151 | Solyc02g087460 | LOC101263367 | AT3G28450 | BIR2 | PPC:1.12.1 |  |  | HopAF1 | HopAI1 |
| 153 | Solyc05g010400 | LOC101250403 | AT5G16000 | NIK1 | PPC:1.12.2 | AvrPto | HopA1 | HopAF1 | HopAI1 |
| 156 | Solyc07g006110 | LOC101254706 | AT2G23950 |  | PPC:1.12.2 | AvrPto | HopA1 | HopAF1 | HopAI1 |
| 160 | Solyc08g081940 | LOC101265354 | AT4G23740 | NbIRK | PPC:1.13.3 | AvrPto | HopA1 | HopAF1 | HopAI1 |
| 161 | Solyc06g068910 | LOC101247929 | AT1G48480 | RKL1 | PPC:1.13.3 |  | HopA1 |  | HopAI1 |
| 163 | Solyc03g095490 | LOC101252259 | AT5G58300 | none | PPC:1.13.3 | AvrPto | HopA1 | HopAF1 | HopAI1 |
| 188 | Solyc02g089900 | LOC101261978 | AT2G23770 | LYK4 | PPC:1.1.3 | AvrPto | HopA1 | HopAF1 | HopAI1 |
| 196 | Solyc10g012170 | LOC101255141 | AT4G00330 | CRCK1-like | PPC:1.5.3 | AvrPto | HopA1 | HopAF1 | HopAI1 |
| 221 | Solyc05g013070 | LOC101259335 | AT1G14000 | None | PPC:2.1.2 | AvrPto | HopA1 | HopAF1 | HopAI1 |
| 250 | Solyc12g010130 | CIPK6 | AT4G30960 | CIPK6 | PPC:4.2.4 |  | HopA1 |  | HopAI1 |
| 255 | Solyc06g068450 | CIPK25 | AT5G25110 | CIPK25 | PPC:4.2.4 |  | HopA1 | HopAF1 |  |
| 259 | Solyc06g008330 | LOC101267610 | AT2G20470 | NDR1 | PPC:4.2.6 |  | HopA1 | HopAF1 | HopAI1 |
| 272 | Solyc12g062870 | LOC101266803 | AT5G14640 | SK13 | PPC:4.5.4 |  | HopA1 | HopAF1 | HopAI1 |
| 279 | Solyc08g074760 | LOC101257290 | AT2G35620 | FEI2 | PPC:1.12.2 |  | HopA1 | HopAF1 | HopAI1 |
| 304 | Solyc03g123800 | LeMKK2 | AT3G21220 | MKK5 | PPC:4.1.3 | AvrPto | HopA1 | HopAF1 | HopAI1 |
| 311 | Solyc03g115700 | LOC543870 | AT3G01090 | KIN10 | PPC:4.2.4 |  | HopA1 |  | HopAI1 |
| 318 | Solyc04g012160 | SRK2C | AT4G33950 | OST1 | PPC:4.2.4 | AvrPto | HopA1 | HopAF1 |  |
| 323 | Solyc06g071210 | LOC101246352 | AT3G08720 | S6K2 | PPC:4.2.6 | AvrPto | HopA1 | HopAF1 | HopAI1 |
| 327 | Solyc12g019460 | LeMPK1 | AT2G43790 | MPK6 | PPC:4.5.1 | AvrPto | HopA1 | HopAF1 | HopAI1 |
| 342 | Solyc10g047140 | SERK3A | AT4G33430 | BAK1 | PPC:1.12.2 |  |  | HopAF1 | HopAI1 |
| 376 | Solyc01g103940 | LOC101248921 | AT1G60940 | SnRK2.10 | PPC:4.2.4 |  | HopA1 | HopAF1 | HopAI1 |
| 339 | Solyc06g069330 | LOC101264833 | AT5G08160 | ATPK3 | PPC:5.1.1 | AvrPto | HopA1 | HopAF1 | HopAI1 |
