# Supplementary material for: Integrative network-centric approach reveals signaling pathways associated with plant resistance and susceptibility to Pseudomonas syringae
Source: PLoS Biol. 2018 Dec 12;16(12):e2005956. doi: 10.1371/journal.pbio.2005956 (PMC6322785; doi:10.1371/journal.pbio.2005956)
Supplement: S3 Table — The table lists the gene lab ID, the sequences of the PCR oligonucleotides used for amplification, the expected size of the PCR amplicon, the gene ID in tomato and Nicotiana benthamiana, the number of targeted genes, and the length of overlap between the target and PCR amplicon. The last two columns list potential off-target genes as well as the maximum length of the off-target regions. The PCR oligonucleotides were tested using the SGN VIGS tool at http://solgenomics.net/tools/vigs. SGN, SolGenomics Network; VIGS, virus-induced gene silencing. (DOCX) [file pbio.2005956.s012.docx]

**STable 3**: VIGS amplicons used in this study. The table lists the gene lab ID, the sequences of the PCR oligonucleotides used for amplification, the expected size of the PCR amplicon, the gene ID in tomato and *Nicotiana benthamiana*, the number of targeted genes and the length of overlap between target and PCR amplicon. The last two columns list potential off-target genes as well-as the maximum length of the off-target regions. The PCR oligonucleotides were designed using the SGN VIGS tool at <http://solgenomics.net/tools/vigs>.

| **Lab ID** | **Tomato Gene ID** | **Left Primer** | **Right Primer** | **Amplicon length (nt.)** | ***N. benthamiana* predicted targets** | **Target overlap (nt.)** | **Number of potential OFF targets** | **Max OFF target overlap (nt.)** |
| --- | --- | --- | --- | --- | --- | --- | --- | --- |
| **KEI104** | Solyc11g064890.1.1 | TGCCTCTGTCTCCACTTGGT | GTGCAATTTGTGCCTCACTTT | 420 | NbS00049297g0008.1; NbS00008710g0016.1 | 194; 186 | 5 | 9 |
| **KEI143** | Solyc02g081070.1.1 | GTAACCGGTCTCCTCCTCGT | ATCGGGGACTTCACTCTCCT | 361 | NbS00055518g0003.1 | 361 | 0 | 0 |
| **KEI149** | Solyc03g006890.1.1 | AAGGGTGGGATGGAAAAGAG | TTCGACTCCGAAATGGGTAG | 155 | NbS00004867g0005.1 | 118 | 0 | 0 |
| **KEI150** | Solyc02g068300.1.1 | ATGGGATTATTGACCGCAAA | TGCAACCCTGCTTTTATTGTT | 297 | NbS00004867g0005.1 | 297 | 0 | 0 |
| **KEI151** | Solyc02g087460.1.1 | CGATTCGGCTAATTTTGAGG | GGCGACCAACAAATCAACTAA | 313 | NbS00030119g0004.1; NbS00010718g0005.1 | 190; 147 | 2 |  |
| **KEI153** | Solyc05g010400.1.1 | TGTTGATCCCTGTAGCTGGA | TTGTGCTTTTGTCTCCACCA | 630 | NbS00012058g0007.1 | 173 | 1 | 33 |
| **KEI156** | Solyc07g006110.1.1 | CAATAACTTGACTGGACCTATTCC | AATTGTTCGTTGCACGTTGA | 254 | NbS00005344g0026.1; NbS00023855g0005.1 | 254 | 0 | 0 |
| **KEI160** | Solyc08g081940.1.1 | TTAGGTTCTTGAAAATGGAGTGG | AGGAATTGGACCATTGAAACC | 275 | NbS00027675g0005.1; NbS00030754g0008.1 | 233; 215 | 0 | 0 |
| **KEI161** | Solyc06g068910.1.1 | CTCATCTTCACCACAACATCG | GAAGTGTTCTCAATCGGGTCA | 454 | NbS00005919g0003.1 | 85 | 0 | 0 |
| **KEI163** | Solyc03g095490.1.1 | CGCTCCCTATTTGCAATTCT | AGATTGAAGGGAAGGGATGG | 211 | NbS00007294g0015.1; NbS00005729g0009.1 | 182; 179 | 2 | 1 |
| **KEI188** | Solyc02g089900.1.1 | TCCTTTGGAGCTTTCTCAGC | CCAACAACACCAACCACAAC | 597 | NbS00052773g0007.1; NbS00008036g0006.1 | 105; 98 | 2 | 3 |
| **KEI196** | Solyc10g012170.1.1 | CCAGCTACATGACCCATTCA | ATACTCCCTGCCTCCTTTCC | 361 | NbS00001871g0010.1; NbS00012422g0010.1 | 170; 121 | 2 | 1 |
| **KEI20** | Solyc01g097980.1.1 | ACTCCTTCCACGACGTCAAC | CCGAGTATTTGACCGTTGCT | 218 | NbS00017432g0014.1; NbS00011737g0001.1 | 403; 314 | 0 | 0 |
| **KEI221** | Solyc05g013070.1.1 | AGTCAGCAAAACATCGCAAA | TGCGATTATCGTAATCCCTAGC | 116 | NbS00002505g0024.1; NbS00017293g0010.1 | 314; 263 | 0 | 0 |
| **KEI25** | Solyc02g078140.1.1 | TGGATGAAGAGGTTAATTCTTGG | TCGGAAATTGCTGTTACTGG | 250 | NbS00033220g0003.1; NbS00035332g0004.1 | 260; 212 | 0 | 0 |
| **KEI255** | Solyc06g068450.1.1 | TGAAAATGCAAGGAACATACG | CTGCCATCATTCTCACCTTG | 181 | NbS00053231g0003.1; NbS00009921g0102.1 | 33; 20 | 0 | 0 |
| **KEI259** | Solyc06g008330.1.1 | TCCAGAAGTTTCAACCACGA | TCATCAGCACCCATTTTGTG | 334 | NbS00002051g0010.1; NbS00023439g0002.1; NbS00006379g0032.1; NbS00025105g0013.1 | 187; 124; 83; 74 | 3 | 9 |
| **KEI272** | Solyc12g062870.1.1 | TGGTGGTATAATTCCTTCAGCA | CTTAGGCTCGCCATTTTTCC | 202 | NbS00003134g0111.1; NbS00000901g0007.1 | 321; 296 | 0 | 0 |
| **KEI279** | Solyc08g074760.1.1 | CAGAAGATTCGGACCCATGT | TTTGCTGGCCACACAAATTA | 465 | NbS00032709g0016.1; NbS00056913g0001.1 | 315; 281 | 2 | 77 |
| **KEI304 (MKK5)** | Solyc03g123800.1.1 | ATGCGACCTCTTCAACCAC | GGGAGGAAGAGGAAGACGAC | 214 | NbS00012713g0030.1; NbS00006609g0002.1 | 49; 27 | 0 | 0 |
| **KEI311** | Solyc03g115700.1.1 | CTTGGCTTCCAGTGGCTATC | TACTTAGGGCAGTCGCATTG | 359 | NbS00001056g0057.1 | 270 | 1 | 125 |
| **KEI318** | Solyc04g012160.1.1 | GAAGGAGAGGAAGCTAGTTTACAA | AATGCGCAAACAAAATCTCC | 212 | NbS00013414g0001.1; NbS00061263g0003.1 | 231; 177 | 2 | 40 |
| **KEI323** | Solyc06g071210.1.1 | AAAAAGGCCCTTCATTCCAT | CCCTGGCCCTATTTTCCTAA | 336 | NbS00015787g0004.1; NbS00003311g0003.1; NbS00054087g0006.1 | 273; 146; 77 | 0 | 0 |
| **KEI327 (MPK6)** | Solyc12g019460.1.1 | GGATGGTCCAGCTCATCAAG | TGCCATAAGCTCCTTTACCG | 218 | NbS00060107g0004.1; NbS00036924g0003.1; NbS00003284g0009.1; NbS00003284g0006.1 | 254; 162; 133; 90 | 0 | 0 |
| **KEI33** | Solyc06g082440.1.1 | GGTGGATGATTTGGAGAGGA | TCAGATGTAGCCACCGGAAT | 313 | NbS00033396g0001.1; NbS00057370g0001.1 | 33; 17 | 0 | 0 |
| **KEI339** | Solyc06g069330.1.1 | ATGGGGTGCTCGTTCTCG | GACCTCCTTCACGAGGAAAA | 148 | NbS00043523g0017.1; NbS00030449g0013.1 | 54; 45 | 0 | 0 |
| **KEI342 (BAK1)** | Solyc10g047140.1.1 | TTGGAAGTTCCTCCAGCTTC | TTCACGCAAGGAAAACCTCT | 252 | NbS00044412g0010.1; NbS00003411g0018.1 | 519; 465 | 0 | 0 |
| **KEI37** | Solyc02g065520 | GGGAGTTCCTCTTGTCTCTTTG | GGCACGAGTAAAAGCTGGTC | 284 | NbS00012824g0002.1; NbS00006810g0118.1 | 112; 86 | 0 | 0 |
| **KEI376** | Solyc01g103940.1.1 | ACAGAACAAGCCCAAGCAGT | TGAAACTCTCCACTAGCATGAA | 254 | NbS00001404g0029.1; NbS00005219g0010.1 | 170; 126 | 2 | 41 |
| **KEI7** | Solyc07g042590 | GCACCAACATAACAAGCCAGT | TGCTTAGTAGACAACTTCATCTCTTCA | 215 | NbS00046910g0007.1; NbS00040223g0007.1 | 252; 163 | 2 | 6 |
| **KEI72 (SOBIR1)** | Solyc06g071810.1.1 | TCTCCTGCCATTGGAAAACT | CAGGCACATGACCTTCAAGA | 364 | NbS00037616g0014.1; NbS00033954g0002.1; NbS00006092g0015.1 | 80; 56; 25 | 3 | 8 |
| **KEI86** | Solyc11g072660.1.1 | TGGGTTGTTTTCCTTGTTCTG | CTTAGCACCGCCAGTTTTTC | 251 | NbS00031691g0018.1; NbS00046018g0001.1;NbS00003259g0009.1; NbS00037822g0005.1 | 132; 132; 69; 38 | 0 | 0 |
| **KEI91** | Solyc03g113450.1.1 | CAGCAAAGTGGCCATAGTGA | GTCGAATTCGGAAAGATCCA | 310 | NbS00042791g0006.1; NbS00049670g0007.1 | 289; 185 | 0 | 0 |
| **KEI92** | Solyc05g056370.1.1 | GTATCCCACCGCAGTTGTTT | TTGGCCTAAGGAAATGTTGC | 281 | NbS00019122g0004.1; NbS00025463g0012.1 | 281 | 0 | 0 |
