# Supplementary material for: Integrative network-centric approach reveals signaling pathways associated with plant resistance and susceptibility to Pseudomonas syringae
Source: PLoS Biol. 2018 Dec 12;16(12):e2005956. doi: 10.1371/journal.pbio.2005956 (PMC6322785; doi:10.1371/journal.pbio.2005956)
Supplement: S1 Materials and Methods — KEI, Kinase Effector Interactor; PCD, programmed cell death; SLCA, split luciferase complementation assay; VIGS, virus-induced gene silencing. (DOCX) [file pbio.2005956.s027.docx]

**Supplemental Methods**

***SLCA data analysis***

We analyzed 1170 pairs of Kinase-Effector interactions, including 210 Kinase-AvrPto pairs, 299 Kinase-HopA1, 305 Kinase-HopAI1, and 326 Kinase- HopAF1, and 30 HopM1-Effector pairs. Data were normalized using four control signals per plate. We employed the following controls: 1) positive control (full Luciferase), 2) negative control (no Luciferase, to assess background luminescence), 3) positive functional control (Pto-AvrPto); 4) functional negative control (Pto-AvrPtoI96A). Raw data from plate reader (BioTek’s Synergy 2 micro-plate reader) is available at https://figshare.com/s/35c4aab65174c67a496e. The dynamics of the fluorescence reaction was capture at six time points, with a 10-minute interval between measurements. We targeted the first data point to the maximum of fluorescence after the addition of substrate (Viviren), which occurs at ~ 8 minutes for the Luciferase only probe; the captured dynamics on controls and probes show variability due to the variation in efficiency of reconstituted NLuc and CLuc enzyme over the substrate. The K-E signal had a large dynamic range (as large as the positive control) indicating that there are no physical limitations in measuring the luminescence produced by our split luciferase probes. Four technical replicates were performed for each pair of effector-target protein interaction. The data were represented as vectors in the 6-dim space of recorded time series. The background correction consists in subtracting the negative control measurements from the luminescence signal of each probe. The time series of the K-E probes data were used to compute the normalized fluorescence intensity signals (L2 norm) referenced to the background level of each plate:

$$\boldsymbol{y}_{N}=sgn\left( \sum_{i=1}^{6} \left( y_{i}-b_{i} \right) \right)\left\| \boldsymbol{y}-\boldsymbol{b} \right\|, \boldsymbol{y}=\left[ y_{1},{\cdots y}_{6} \right],$$

$$\boldsymbol{y}:KE probe signal, \boldsymbol{b}:background$$

The normalized control signal was similarly computed with:

$$\boldsymbol{c}_{N}=sgn\left( \sum_{i=1}^{6} \left( c_{i}-b_{i} \right) \right)\left\| \boldsymbol{c}-\boldsymbol{b} \right\|, \boldsymbol{c}=\left[ c_{1},{\cdots c}_{6} \right]$$

$$\boldsymbol{c}:functional negative control,\boldsymbol{b:}background$$

Known interactions (Pto-AvrPto, and Pto-AvrPto^I96A^) were used for data normalization and testing of kinase-effector interactions. To account for samples biological variation, we normalized the data using multiple regression. We developed a regression model where the inhibited interaction level (Pto-AvrPto^I96A^) of each plate depends on the level of the positive functional control (Pto-AvrPto) and luciferase (Luc) signals: $\boldsymbol{c}_{Pto-AvrPtoI96A}\sim{(\boldsymbol{c}}_{Pto-AvrPto}, \boldsymbol{c}_{Luc )}$ (R-squared: 0.887, Adjusted R-Squared 0.886) (**S1D Fig)**. We used the model to predict the level of normalized inhibited signal level $\boldsymbol{c}_{I}$ by fitting a linear model with using the positive control and luciferase signals in each plate. We then tested for K-E interactions significantly stronger than the level of normalized inhibited interaction in each plate.

We used a one-sided t-test: $t-test=\frac{\bar{\boldsymbol{y}_{N}}\boldsymbol{-}\bar{\boldsymbol{c}_{I}}}{{s_{p}}/\sqrt{2}},s_{p}:pooled standard deviation$ with the alternative hypothesis: *probe’s normalized signal is greater than the normalized inhibited control signal* at the 5% significance level. To control error rate in our multiple testing of K-E interactions, we applied the FDR method to the kinase-effector testing results. We calculated a relative interaction strength (fold change) as the ratio of mean KE probe signal to the mean of normalized inhibited signal: $\delta=\bar{\boldsymbol{y}_{N}}\boldsymbol{/}\bar{\boldsymbol{c}_{I}}$. We used Cytoscape [1] to represent the resulting K-E interactome and for visualization of quantitative measures of the interactions. The data processing, matrix calculation, normalization, and testing was implemented in a MATLAB script [2] (**S3 Data**).

***Statistical testing of bacterial growth and PCD assays for VIGS silenced KEIs***

We used Student’s 2-tailed t-test for bacterial growth assays at significance level 0.05, and the Wilcoxon rank-sum test for MKK7ox, MKK9ox, and HopQ1-1 PCD assays to test for significant phenotype changes between control and *KEI*-silenced lines. The *KEIs* with significantly different phenotype response (pValue < 0.05) were selected as components of kinase-effector interactome and were used for the inference of immune response networks. Two phenotypes (bacterial growth and PCD) were quantified as described above; the effectors tested using bacterial growth assays were grouped into PTI-associated (D29E-effectorless) and ETS-associated (HopA1, HopAI1, HopAF1, AvrPto); PCD quantification was used to test the HopQ1-1 challenge response (identification of ETI-associated pathways) and the effect of MKK7 and MKK9 overexpression in the background of silenced KEIs. The combinations of effectors (D29E-effectorless, HopA1, HopAI1, HopAF1, AvrPto, and HopQ1-1) and *KEI*s were used as an indicator that the silenced *KEIs* function in the effector response pathway. The combinations of overexpressed *MKK* (Mkk7ox, Mkk9ox) and silenced *KEIs* were used as an indicator that the *KEIs* function in the signaling pathways involving the MKK7/9. We tested 200 (40 kinases x 5 effectors) combinations of silenced *KEIs* and effectors (**S1 Data. Bacterial growth assays - data**):

$$test\left( \left\{ Ki,e \right\} \right);$$

$$Ki\in\left\{ \begin{aligned} Ki7, Ki20,Ki25, Ki33,Ki37,Ki72,Ki86,Ki91,Ki92,Ki104,Ki143,Ki146, Ki149,Ki150,Ki151,Ki153, \\ Ki156,Ki160,Ki161,Ki163,Ki188,Ki196,Ki221,Ki245,Ki250,Ki255,Ki259,Ki272,Ki279,Ki304, \\ Ki311, Ki315,Ki318,Ki323,Ki327,Ki339,Ki342,Ki376,Ki377,AtFLS2 \end{aligned} \right\}$$

$eff\in\left\{ D29E, HopA1,HopAI1,HopAF1, AvrPto \right\}$,

36 combinations (including AtFLS2) of silenced KEIs with the *HopQ1-1* effector (**S1 Data.** **Cell death treatments - data**):

$test\left( \left\{ Ki,eff \right\} \right);$ $Ki\in\left\{ Ki7, \ldots,AtFLS2 \right\};eff\in\left\{ HopQ1-1 \right\}$

and 72 combinations of silenced KEIs and MKK overexpression:

$test\left( \left\{ Ki,KKox \right\} \right);Ki\in\left\{ Ki7, \ldots,AtFLS2 \right\};KKox\in\left\{ MKK7ox, MKK9ox \right\}$ ,

quantifying independently the phenotype for each of the assays: bacterial growth and PCD.

${PhQ}_{assay}\left( Ki,s_{assay} \right);assay\in\left\{ BG, PCD \right\};Ki\in\left\{ Ki7, \ldots,AtFLS2 \right\};$ with

$s_{BG}\in\left\{ D29E, HopA1,HopAI1,HopAF1, AvrPto \right\}; s_{PCD}\in\left\{ HopQ1-1,MKK7ox, MKK9ox \right\}$ ,

using series of measurements as described in the “Bacterial growth assays” and “PCD assays” sections.

***Correlation of immune responses (intensity of phenotypes) for KEIs with significant stress phenotype***

We performed a correlation analysis using the Pearson's correlation coefficient for KEIs with significant stress phenotype. We computed the intensity of phenotype *I (s,Ki)* for each stress assay and tested KEIs as the median values over the series of observations, normalized by the median of control for each assay. The correlation coefficients were computed over the common sets of KEIs for the eight stress assays:

$s\in\{D29E, HopA1,HopAI1, HopAF1, AvrPto, HopQ1\text{-}1, MKK7ox, MKK9ox\}$.

The matrix of correlation coefficients of measured phenotypes is defined as $PhQ\_corr(s1,s2)=corrcoef(\vec{I_{s1}},\vec{I_{s2}})$. The coefficients indicate the degree of correlation for the observed phenotypes of pairs of pathogen effectors or overexpressed KK over the sets of kinases that significantly affect these phenotypes.

***Co-occurrence pattern analysis for immune pathways inference***

We generated a ‘KEI vs. treatment’ matrix (named here ‘network effects’ matrix) that was subsequently used for the inference of KEI network structure subject to a set of structural constraints of signaling pathways. The network effects matrix (with 36 rows and eight columns) is a Boolean matrix containing the decisions of the bacterial growth and PCD assay statistical tests. An entry of “1” indicates whether that combination of effector-kinase or overexpressed KK – kinase has a significant effect on the measured phenotype. The constraints were derived from the functional classification and cellular localization of analyzed kinases, and from the known structure of conserved signaling pathways. Thus, we defined four levels of kinases signaling architecture: 1. Receptor-like kinases (RLKs), 2. Receptor-like cytoplasmic kinases (RLCKs), 3. Mitogen-activated protein kinases (including MAP3Ks-Rafs, MAP2Ks, and MAPKs), and 4. Other cytosolic kinases (OCKs), including SnRKs, AGCs, S6K-like, GSK3-like. The analyzed set of kinases includes 16 RLKs, 5 RLCKs, 5 MAPKs, and 10 OCKs. We made further assumptions about the structure of the signaling pathways, preserving the canonical structure of MAPK cascades (MAP3Ks/RAFs->MKKs->MAPKs) and constraining the signal transduction to follow the direction from the first to the fourth level. Next, we developed an analysis method of patterns of co-occurrence to infer the signaling pathways from the combinatorial kinase silencing and treatments (BG and PCD).

The network construction algorithm is described below:

----------------------------------------------------------------------------------------------------------------

**Algorithm: *Co-occurrence network***

----------------------------------------------------------------------------------------------------------------

*Input*: $V=\left\{ K_{i} \right\}; L\left( K_{i} \right)\in\left\{ RLK,RLCK,MAPK,OCK \right\},i=1..N;E_{init};S_{j}\in\left\{ D29E,\ldots MKK9ox \right\};j=1..M; PhE\left[ NxM \right],NE\left[ NxM \right];$

*Output*:$G\left( V,E \right);M_{cooc}\left[ NxN \right];M_{cooc}\left( i,k \right)=\sum_{j=1..M} \left( NE(i)\bigwedge NE(k) \right)$

$1. E=E_{init};$ ${Compute M}_{cooc}$.

$Repeat to convergence$:

$$\{$$

$$2. Network grow$$

$For l$=1..3

$$For each K_{i} in L(l)$$

$$For each K_{j} in L\left( l+1 \right)$$

$$if \left( M_{cooc}\left( i,j \right)\geq T_{1} and S(PhE\left( i,: \right),PhE\left( j,: \right))\geq T_{2} \right) add edge e(K_{i},K_{j})$$

end

$$end$$

$$end$$

$$3. Network trim$$

$For l$=1..4

$$For each K_{i} in L(l)$$

$For each K_{j} in L\left( l \right),K_{i}\neq K_{j}$

$$if \left( S\left( NE\left( i,: \right),NE\left( j,: \right) \right)\geq T_{3} \right) add edge e(K_{i},K_{j})$$

$$if (O_{i}\left( NE\left( i,: \right),NE\left( j,: \right))\geq O_{j}(NE\left( i,: \right),NE\left( j,: \right)) xor l>2 \right)$$

$remove all edges from K_{i} to the next layer nodes shared with K_{j}$

$$remove all edges from K_{j} to the previous layer nodes shared with K_{i}$$

else

$remove all edges from K_{j} to the next layer nodes shared with K_{i}$

$$remove all edges from K_{i} to the previous layer nodes shared with K_{j}$$

*end*

*end*

$$end$$

$$end$$

*end*

*Adjust* $T_{1}, T_{2} ,T_{3}$*.*

*}*

$$4. Network compete$$

/*add skip layer edges for high node co-occurrence patterns*/

$${if (K_{i} and K_{i} in non adjacent layers and S\left( NE\left( i,: \right),NE\left( j,: \right) \right)\geq T_{5}) add edge e(K_{i},K_{j})}_{.}$$

/*add skip layer edges for isolated nodes*/

$${if \left( {degree(K}_{i} \right)=0, K_{j} in non adjacent layer and S\left( PhE\left( i,: \right),PhE\left( j,: \right) \right)\geq T_{6}) add edge e(K_{i},K_{j})}_{.}$$

----------------------------------------------------------------------------------------------------------------

Post construction network analysis and visualization:

1. *Build* an effector-specific networks using the $-log(pVal)$over edges to map interaction strength.

2. *Create* SSN signed networks with *positive* or *negative* effect on the measured phenotype using the *phenotype effects* matrix.

The networks were visualized in Cytoscape [1]. Calculations were implemented using a MATLAB script [2].

***KEI signaling network analysis***

We used Cystoscope network plugins to perform the KEI signaling network analysis. Network parameters (including shortest path, diameter, clustering coefficient) were calculated using the NetworkAnalyzer [4] for each effector specific and PCD assay network. Histograms of path lengths for each SSN were compared. “Essential” network nodes were identified using the MCC clique analysis method [5]. The calculation were carried on independently for the PTI network, and ETS aggregated networks. PTI and ETS nodes were ranked based on Maximal Clique Centrality scores. Critical networks of top 10 highest MCC ranked nodes were computed for both PTI and ETS networks. Signed stimulus-specific networks (SSN) were computed from the inferred KEI network. The sign represents the effect of the KEI on assay: positive/virulence, negative/defense. Distribution patterns of signed nodes were analyzed for each class of kinases.

A weighted interaction network of Arabidopsis homologs of *KEIs* based on curated associations downloaded from STRING was computed in **Fig 6F**. Edges represent protein-protein interactions (PPI) (continuous lines) or co-expression (interrupted lines). Edge weights are proportional with confidence scores calculated in STRING v10, scaled between zero and one, and indicating the likelihood that interactions are biologically meaningful, specific, and reproducible. Edge labels indicate the signal-specific networks in which connected KEIs are co-occurring. The network was uploaded in Cytoscape v3.6.1 for visualization.

**References:**

1. Shannon P, Markiel A, Ozier O, Baliga NS, Wang JT, Ramage D, Amin N, Schwikowski B, Ideker T., *Cytoscape: a software environment for integrated models of biomolecular interaction networks*. Genome Res. 2003 Nov;13(11):2498-504.

2. The MathWorks, Inc., *MATLAB and Statistics Toolbox*. Release 2015, Natick, Massachusetts, United States.

3. Sievers F, Wilm A, Dineen D, Gibson TJ, Karplus K, Li W, Lopez R, McWilliam H, Remmert M, Söding J, Thompson JD, Higgins DG., *Fast, scalable generation of high-quality protein multiple sequence alignments using Clustal Omega*. Mol Syst Biol. 2011 Oct 11;7:539. doi: 10.1038/msb.2011.75.

4. Doncheva NT, Assenov Y, Domingues FS, Albrecht M., *Topological analysis and interactive visualization of biological networks and protein structures*. Nat Protoc. 2012 Mar 15;7(4):670-85. doi: 10.1038/nprot.2012.004.

5. Chin CH, Chen SH, Wu HH, Ho CW, Ko MT, Lin CY., *cytoHubba: identifying hub objects and sub-networks from complex interactome*. BMC Syst Biol. 2014;8 Suppl 4:S11. doi: 10.1186/1752-0509-8-S4-S11. Epub 2014 Dec 8.
